# When does forecast-based insurance benefit? An economic analysis of drought risk anticipatory insurance

**DOI:** 10.1057/s41288-025-00355-2

**Published:** 2025-08-24

**Authors:** Vaibhav Anand, Leah Poole-Selters, Alexa Gozdiff Spognardi, Biniam Taddese Bekele, Erin Coughlan de Perez

**Affiliations:** 1 Greenberg School of Risk Management, Tobin College of Business, St. John’s University, New York, USA; 2 Feinstein International Center, Tufts University, Medford, USA; 3 Tufts University, Medford, USA; 4 African Risk Capacity, Johannesburg, South Africa; 5 Friedman School of Nutrition Science and Policy, Tufts University, Medford, USA

**Keywords:** Forecast-based insurance, Drought risk, Anticipatory insurance, Index insurance, Economic analysis, Disaster finance

## Abstract

Improvements in forecasting technologies create opportunities for anticipatory actions before disasters occur. However, traditional *ex post* financing and limited operational capacity often prevent countries from acting early. This paper examines anticipatory index insurance, specifically African Risk Capacity’s pilot program in Malawi and Zambia for drought risk, which offers capacity building and forecast-based financing for early actions. However, its benefits are unclear given the trade-off between early actions based on imperfect forecasts and post-disaster relief after certain losses. Since imperfect forecasts increase basis risk, anticipatory index insurance can exacerbate this trade-off. Using a stylized economic model and numerical analysis, we identify conditions under which anticipatory insurance is beneficial. Results show that its primary value lies in building operational capacity for forecast-based actions. However, due to basis risk, the incremental value from insurance payouts may not always justify the premium costs. Our findings can help governments and aid agencies design optimal financing strategies for anticipatory actions.

## Introduction

Climate change is disrupting precipitation patterns, leading to more frequent droughts, which can harm agricultural productivity and threaten food security (IPCC 2021). Recent advances in seasonal weather forecasts provide an opportunity to develop forecast-based early action plans that enable predetermined actions when forecasts exceed a trigger (Coughlan de Perez et al. 2015).^1^ These plans can reduce the potential negative consequences of drought at a lower cost by acting early before the disaster (Aguirre et al. 2019; Gros et al. 2023). However, the effectiveness of these programs depends on having a reliable early-action capacity, which involves coordinating across various institutions, establishing standard operating procedures, mapping vulnerable populations, training personnel, and allocating pre-disaster funding promptly for early actions. Given the traditional reliance on ex post financing model for disaster recovery, it is often challenging to develop this early action capacity and access financing for early actions (Braman et al. 2013; Clarke and Dercon 2016; Lopez et al. 2020).

This paper examines whether a forecast-based anticipatory insurance product can address these challenges. This mechanism merges the well-studied concept of index insurance with the emerging practice of forecast-based early-action plans. It uses forecasts of risk as a trigger for insurance payouts that fund predetermined actions before the risky event occurs. We focus on a pilot anticipatory insurance product designed by African Risk Capacity (ARC) to be piloted in Zambia and Malawi, providing payouts when drought forecasts exceed a predetermined trigger (ARC 2023). The payouts finance specific early actions and support capacity building for their implementation. ARC collaborates with local governments to offer training, establish institutional frameworks, standard operating procedures, and guidelines to enable effective implementation and management of anticipatory insurance-funded early actions.^2^

The anticipatory insurance product has two primary advantages. First, it provides financing through insurance payouts at critical times when drought is likely. Second, it builds early action capacity, enabling countries to mitigate losses at lower costs. For example, access to drought forecasts and timely funding allows farmers to plant drought-resistant crops before the planting season. A country may have forecasts and funds but lack mechanisms to distribute resources or procure seeds quickly, limiting effectiveness. Other capacity challenges may include limited access to forecasts, lack of standard procedures, insufficient trained staff, weak institutional frameworks, inadequate liquidity, or low political will to allocate resources. Anticipatory insurance can address these issues through direct support and evidence-based advocacy. However, this index-based insurance also entails costs and associated risks.

Index insurance is a useful tool for risk financing and offers several advantages over traditional insurance, such as lower cost, faster payouts, and scalability (Linnerooth-Bayer et al. 2005; Barnett and Mahul 2007; Collier et al. 2009). However, it is not without its challenges that has often resulted in low uptake in practice (Giné et al. 2008; Cole et al. 2013). A well-documented problem is the basis risk (Clarke 2016), i.e., insurance payouts may not be correlated with realized losses of the insured, resulting in situations where the policyholder experiences a loss but does not receive a payout or vice versa. The basis risk increases when the underlying index does not provide an accurate indicator of the realized losses (Teh and Woolnough 2019). Although forecasts have improved in recent years (Bauer et al. 2015), there are significant inequities in forecast quality across lead times, risks, and geographies (Linsenmeier and Shrader 2023).

The relative value of anticipatory insurance also depends on the effectiveness of underlying early actions and a country’s early action capacity.^3^ For example, when communities already have sufficient capacity for forecast-based actions, the incremental benefit of anticipatory insurance may come solely from its insurance mechanism. These interactions between forecast quality, early action effectiveness, and capacity raise questions about optimal usage and welfare gains. When is anticipatory insurance an appropriate financing mechanism? Should resources be directed toward designing and promoting such products, or should we wait for improved forecasts and prioritize traditional financing mechanisms? Given existing forecasts and early actions, could pure capacity-building support sometimes be more beneficial than anticipatory insurance?

In this paper, we use a stylized model to examine these questions in the context of an anticipatory insurance product designed for drought risk and use numerical analysis to identify economic conditions under which such a product is beneficial. In order to identify the conditions under which this anticipatory insurance product adds value, it is important to know the relative benefits of capacity building and insurance mechanism. In this paper, we show how these relative benefits depend on both the economic conditions prevalent and a country’s current state of early action capacity. The economic conditions that we focus on in this paper are the cost-benefit profile of early actions, forecast skill, the targeted return period of funding, and insurance premium loading.

First, we quantify optimal demand and benefits of anticipatory insurance through numerical analysis under specific assumptions. We identify ranges of forecast skill, benefit-to-cost ratios of early actions, and insurance payout frequencies for which anticipatory insurance is valuable compared to counterfactual strategies. We analyze two counterfactuals based on a country’s existing capacity to self-finance anticipatory actions. In the first, the country lacks capacity for forecast-based early actions and can only build it by purchasing anticipatory insurance. In the second, the country already has capacity, thus can perform forecast-based actions without insurance, likely already doing so to some extent. These counterfactuals help isolate the benefits derived from building capacity versus the insurance mechanism itself. Specifically, we examine two questions:

- What is the benefit of forecast-based anticipatory insurance to a country that lacks the capacity to take anticipatory action, relative to a country that already has the capacity and takes some level of anticipatory actions?
- What are the relative benefits of capacity building and insurance mechanisms of the anticipatory insurance product to a country that lacks the capacity to take anticipatory actions?

Our methodology also combines theoretical economic analysis with qualitative insights from the field. Qualitative analysis includes reviewing the documents and operational workflows of the anticipatory insurance program and interviewing a diverse range of stakeholders. We conducted interviews with ARC product designers, humanitarian partners, local country representatives, and other participants to validate our assumptions and gain insights into the current conditions and constraints they face in implementing forecast-based early actions.

Our analysis provides two main insights. First, the anticipatory product offers greater benefits to countries lacking early-action capacity compared to those already having capacity. Comparing welfare gains, we find most of the anticipatory insurance benefit comes from capacity building, with smaller gains from its insurance mechanism. Numerical results show that optimal demand for anticipatory insurance is generally positive when a country lacks capacity. However, for countries already having capacity, anticipatory insurance demand is positive only under specific conditions—when early actions yield lower benefits relative to costs and forecast quality is high, making it more beneficial to buy index insurance than implement less-effective early actions. Second, we find that anticipatory insurance adds value primarily by building capacity for forecast-based early action. Comparing welfare gains from capacity building alone versus those from anticipatory insurance alone, we show that standalone capacity-building support can be more valuable than bundled support with anticipatory insurance. Although the anticipatory product includes capacity building, insurance premium costs may exceed the incremental benefits from its financing mechanism.

Our qualitative analysis also suggests that institutional and operational capacity is necessary for successful forecast-based early action programs. While many communities already have early-action, safety-net transfer, or disaster recovery programs, these may not adequately support the entire chain of time-sensitive anticipatory actions. When such capacity is absent, anticipatory insurance can help countries quickly build it. The product’s value is particularly notable when there are frictions in allocating funds for uncertain early-action benefits, especially when decision-makers prefer funding actions with tangible, certain outcomes (e.g., disaster recovery). In these cases, standalone capacity-building may not initiate forecast-based actions. Anticipatory insurance is especially useful here, offering immediate financing and capacity building to demonstrate the benefits of early actions, thus fostering political willingness to support forecast-based actions long-term.

Our analysis unpacks the sources of benefits of the anticipatory insurance and provides the decision-makers with a clear guide to evaluate the value of the product for their specific situation. This paper does not provide a one-size-fits-all recommendation on whether to finance anticipatory actions using insurance. Decision-makers should assess their current conditions and use the results in this paper to determine whether the economic conditions are in a range where the anticipatory insurance is the right product for them.

This study contributes to the growing literature on the role of forecasts in adaptation to climate change-related risks (Shrader 2023; Molina and Rudik 2022; Anand 2025), with a particular focus on agricultural applications (Rosenzweig and Udry 2019; Burlig et al. 2024). These studies show that farmers and firms use forecasts to make adaptation decisions during adverse weather conditions. We examine the optimal way to financing these anticipatory adaptations and explore the role of forecasts in designing such financing mechanisms. In this respect, this paper is closely related to the niche literature on forecast-based anticipatory actions (Coughlan de Perez et al. 2015; Lopez et al. 2020; Lala et al. 2021; Anand 2022), which examines the optimal design of financing anticipatory actions. While this literature primarily focuses on the properties optimal design of forecast-based actions, our paper examines whether and under what conditions these actions can be financed through an insurance mechanism. We also contribute to the literature on index-based insurance and weather derivatives. Following the economic framework in Clarke and Ruth Vargas (2013), which examines ARC’s traditional index insurance product, our paper extends the analysis in two primary ways. First, we explicitly incorporate early-action benefits into our model, allowing us to analyze the joint effects of forecast skill and early-action benefits on optimal insurance coverage. Second, we separate welfare gains from anticipatory insurance into capacity building and insurance mechanisms, identifying when each is relatively more beneficial. A sizeable literature examines the benefits (Linnerooth-Bayer et al. 2005; Barnett and Mahul 2007; Collier et al. 2009) and limitations (Giné et al. 2008; Cole et al. 2013; Clarke 2016) of index-based products in managing weather risk. Chantarat et al. (2013) and Teh and Woolnough (2019) evaluate indices for reducing basis risk. Prior research on forecast-based index insurance, such as Khalil et al. (2007), Mortensen and Block (2018), and Collier (2020), focuses on El Niño-Southern Oscillation (ENSO)-based products in Peru but does not examine forecast-triggered early actions. Our paper extends this literature by analyzing forecast-based insurance specifically designed for anticipatory actions, exploring welfare gains under varying forecast skills and early-action effectiveness. Given increasing interest in alternative financing mechanisms for anticipatory actions, our analysis offers practical insights and guidance to investors, aid agencies, and countries about economic conditions where forecast-based insurance is beneficial.

The paper is structured as follows. Section 2 provides the background on the anticipatory insurance product. Section 3 presents the economic analysis model and results. Section 4 discusses the qualitative analysis. Section 5 concludes.

### Forecast-based anticipatory insurance

Malawi and Zambia heavily rely on agriculture, contributing more than 25% and 19% to their GDP, respectively, with significant populations depending on small-scale farming (ReliefWeb 2023). Both countries face persistent food insecurity, affecting around 16 million Malawians to varying degrees and 1.35 million Zambians classified as severely food insecure, requiring immediate humanitarian aid (World Bank 2021). To strengthen disaster risk management, both countries have developed Disaster Risk-Financing Strategies, partnering with humanitarian organizations and development finance institutions, such as the World Bank, to implement index-based insurance and forecast-based early action schemes. One key initiative is the sovereign drought insurance program managed by the African Risk Capacity (ARC).

**Fig. 1:**
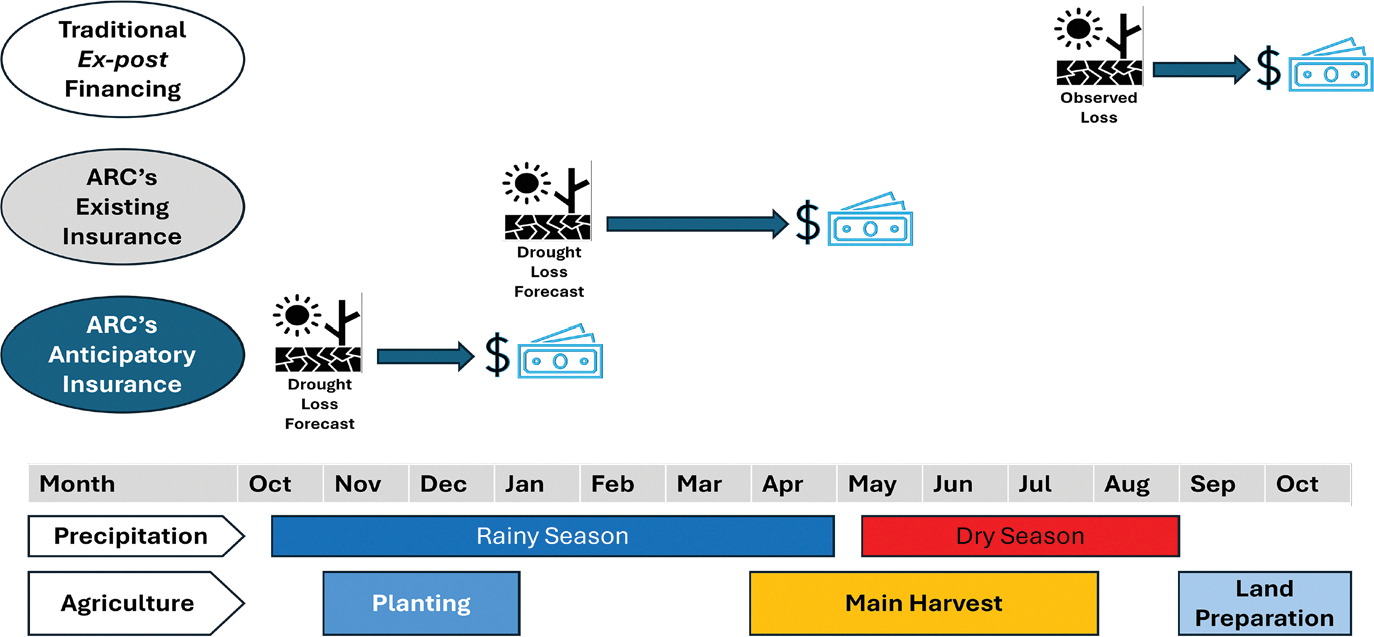
Anticipatory insurance provides payout before the end of the rainy season. *Notes* this figure shows three disaster financing approaches and their timing relative to agricultural seasons in a typical year. It illustrates how anticipatory insurance aligns with the agricultural cycle, providing payouts earlier, allowing for earlier interventions before the rainy season ends. The top row shows traditional ex post financing, which provides funds after an observed loss occurs. The middle row depicts ARC’s existing insurance product, which releases funds based on drought loss forecasts, but still after the drought has begun and close to the dry season. The bottom row represents ARC’s anticipatory insurance product, which provides funds earlier

The ARC Group operates as an entity within the African Union, supporting governments in responding effectively to extreme weather events and natural disasters. ARC assists African governments with proactive planning, preparedness, and rapid response capabilities. This paper focuses on ARC’s drought insurance product, specifically its pilot anticipatory insurance that provides payouts based on drought forecasts before the rainy season ends. ARC uses proprietary software to assess drought likelihood using early-season rainfall data. This anticipatory insurance complements ARC’s existing traditional drought insurance, which provides payouts only after the rainy season. Figure 1 illustrates payout timing for ARC’s existing and anticipatory insurance products relative to agricultural seasons in a typical year. It also shows traditional *ex post* relief financing, highlighting how anticipatory insurance aligns better with agricultural cycles by enabling earlier interventions.

**Fig. 2:**
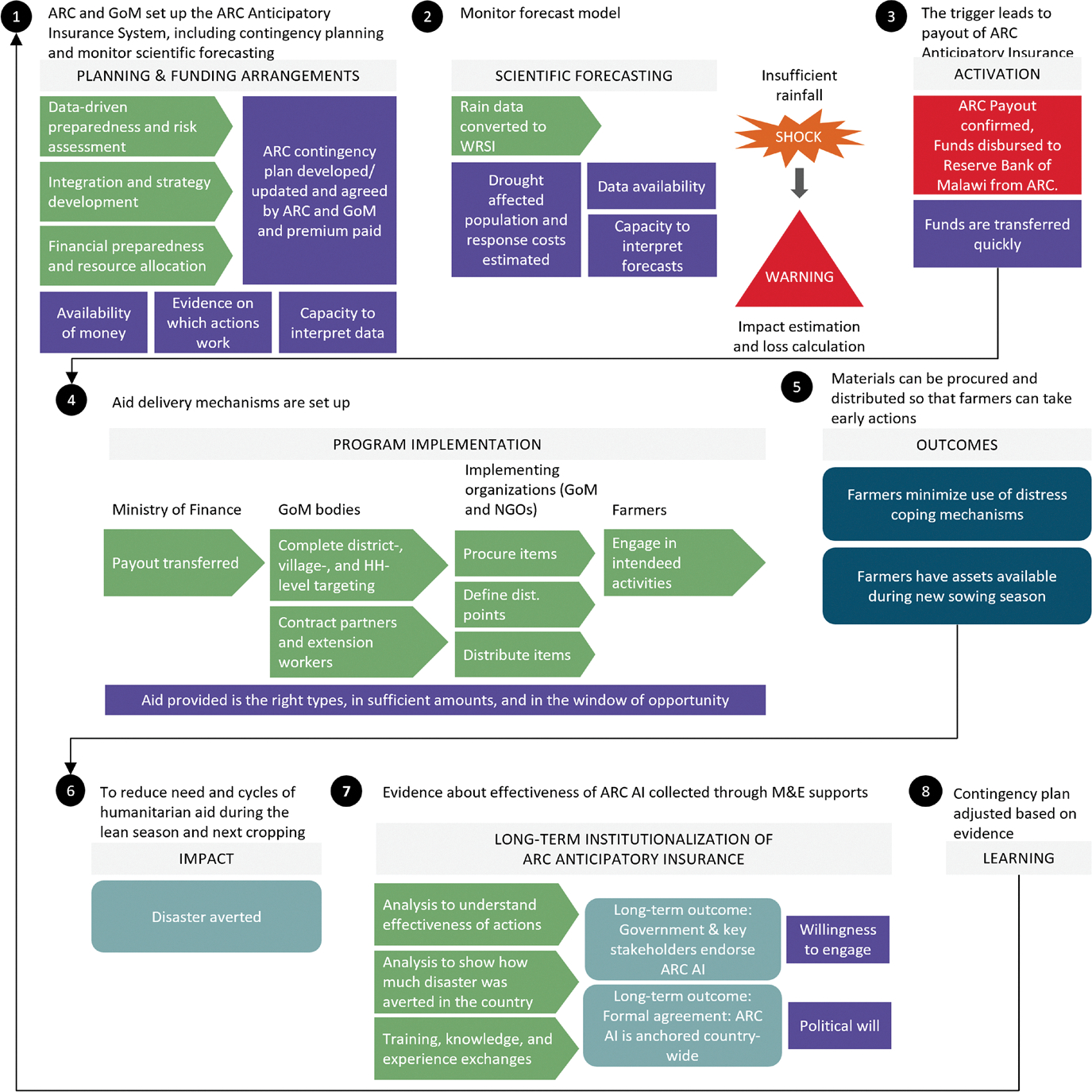
Theory of Change for the anticipatory insurance product in Malawi. *Notes* the figure presents a theory of change flowchart for African Risk Capacity’s (ARC) anticipatory insurance (AI) product’s implementation in Malawi. The figure shows the role played by country’s group of ministers (GoM), ARC, and other non-government organizations (NGOs)

The anticipatory insurance provides payouts before the growing season ends, enabling farmers to take early actions within the ongoing agricultural season. In practice, payouts are transferred by the government to local authorities and partners, who procure and distribute supplies to farmers and households. Figure 2 illustrates the role of various government bodies in program implementation. Early financing during predicted droughts allows farmers to invest in replanting, winter cropping, or small-scale irrigation equipment (ARC 2023). These actions help farmers avoid negative coping strategies, such as reducing consumption or selling productive assets. The anticipatory insurance is accompanied by an early action operating plan detailing specific actions, beneficiary selection criteria, fund distribution processes, and stakeholder responsibilities.

The anticipatory insurance is an index-based product paying out when drought forecasts exceed a predetermined trigger. The index developed by ARC is based on forecasted drought losses. Policy-holding countries select two key parameters: coverage ratio and loss attachment point. When forecasted losses exceed the attachment point, payouts equal the difference multiplied by the coverage ratio. Lower attachment points trigger more frequent payouts. ARC collaborates closely with countries to select suitable parameters based on their needs. Our economic analysis begins by characterizing optimal coverage ratios for different attachment points.

### Economic analysis

In this paper, we use a mixed-method approach to evaluate the forecast-based anticipatory insurance product. We first develop a stylized economic model. We inform our model assumptions and parameters based on the structure and operating plans of the pilot anticipatory insurance product developed by the ARC group. Using this model, we estimate the optimal levels of insurance and early action for both anticipatory insurance scenarios and counterfactual situations. Through numerical simulations, we identify conditions under which this product leads to welfare improvements. Finally, we use qualitative interviews and document analyses to assess whether these optimal conditions are realized in practice.

### Model setup

To assess the conditions under which an anticipatory product could result in a positive economic return and welfare benefit, we build a stylized model based on the canonical economic theory of demand for insurance and early action in Ehrlich and Becker (1972). In our model, a country is endowed with a wealth of w and faces a risk of drought at T=2, which results in loss l. The unconditional probability of drought each year is p. It is the static probability of drought based on the long run climatology. The decision-maker mirrors the risk preferences of country’s residents who have risk-averse preferences over the eventual wealth in period T=2.

### The anticipatory insurance product

The country can purchase an anticipatory insurance product at T=0 that provides trigger-based funds as well as capacity-building support to take forecast-based early actions. Before the realization of the drought, the country receives a drought forecast at T=1, which is the updated probability of drought, ϕ, in that year.^4^ The anticipatory insurance provides a payout s if this forecast probability exceeds a predetermined trigger, the attachment probability $\phi_{\text{att}}$. The probable payment frequency of the anticipatory insurance is once in every 1/q years, where $q=Prob\left(\phi >\phi_{\text{att}}\right)$. The participating country chooses the coverage amount s and the probable payment frequency in years, 1/q, to maximize its utility in period T=2. The choice of payment frequency pins down the attachment probability, $\phi_{\text{att}}$. In practice, the probability distribution of forecasts is likely to be known. In our model, we make the payment frequency, 1/q, a choice variable, which allows us to not make explicit assumptions about the forecast distributions.

The insurance payout s finances a set of predetermined early actions at T=1. These anticipatory actions result in a benefit of β per dollar of investment, in the form of reduction in eventual losses when a drought occurs at T=2, i.e., the loss net of early action benefits is max(l−βs,0). We assume the benefit-to-cost ratio of early action is β>1 when the drought occurs. When the drought does not occur, the benefit-to-cost ratio is 1.^5^

In our model, the amount of insurance is a choice variable, i.e., given the drought frequency and severity in a country, the decision-makers choose the optimal insurance coverage s. Letting insurance coverage be a choice variable allows us to directly examine the effect of economic parameters in the optimal insurance coverage decision. In practice, while the drought probability is often modeled within a close range, every four to five years in our case, actual insurance payouts might vary with drought severity, which often ranges from mild to severe impact on response costs. However, we examine optimal coverage and welfare gains for a given severity of droughts, not a given distribution of severity. This is a limitation of our model. We address this limitation by performing sensitivity analysis of our results to different values of drought probability and severity in Sect. 3.6.^6^

### Basis risk

The forecast of drought is not perfect, which results in basis risk. We assume there is a positive correlation ρ between the occurrence of the drought and the insurance payout. The basis risk materializes when the insurance payout does not trigger, but the drought occurs, or when the insurance payout triggers, but the drought does not occur. Following the parameterization in Clarke (2016) and Clarke and Ruth Vargas (2013), we can express the joint probabilities in our setup as shown in Table 1. Column 1 (column 2) represents the states when the forecast does not exceed (exceeds) the threshold. Row 1 (Row 2) represents the states when drought does not occur (occurs).

The four joint probabilities are {1−q−r,q+r−p,r,p−r}, where q is the probability that the insurance payout is triggered, i.e., $q=Prob\left(\phi \geq \phi_{\text{att}}\right)$ in a year. Note that the return period of the insurance payout is 1/q, that is, the insurance pays every 1/q years on average, which determines the attachment forecast, $\phi_{\text{att}}$. The basis risk is defined by r, which is a function of the return period of insurance payout (which a country can choose) and the correlation between drought and insurance payout (which depends on the forecast technology) such that

(1) $$\rho =\frac{p\;-\;r\;-\;pq}{\sqrt{p(1\;-\;p)q(1\;-\;q)}}.$$

In our analysis, we examine the optimal coverage and utility from various decisions for a range of values of the correlation ρ. This allows us to examine the effect of forecast technology (skill) on an optimal financing strategy.

### Counterfactual scenarios based on country’s capacity

We evaluate anticipatory insurance using two hypothetical scenarios based on a country’s existing capacity for forecast-based early actions. The anticipatory product provides both capacity-building support and an insurance mechanism, with its advantages depending on a country’s current preparedness. Figure 2 details the steps required to design and implement an anticipatory insurance program, highlighting the importance of operational and institutional capacity. On one extreme, some countries may already have sufficient infrastructure, trained personnel, financial resources, and standard procedures. At the other extreme, countries might entirely lack operational and financial capabilities. We analyze anticipatory insurance under these two extremes, recognizing most countries lie somewhere in between.

### Counterfactual 1: Country has no capacity for forecast-based early action

In the first counterfactual, the country lacks operational and financial capacity to independently take forecast-based early actions. Operational constraints may include insufficient trained personnel or lack of a coordinating institution, while financial constraints may involve limited budget allocations or unwillingness to use sovereign funds. Consequently, without anticipatory insurance—which provides both capacity-building support and funds—the country undertakes no forecast-based actions. It relies entirely on anticipatory insurance for early actions. Under this scenario, the decision-maker selects optimal insurance coverage s and insurance payout frequency 1/q to maximize utility $U^{\left(1\right)}$:

(2) $$U^{\left(1\right)}=(1-q-r)\cdot u(w-\pi )+(q+r-p)\cdot u(w-\pi +s)\;\;+r\cdot u\left(w-\pi -l\right)+\left(p-r\right)\cdot u\left(w-\pi -l+\beta s\right),$$

where π is the insurance premium, which depends on coverage s, the return period 1/q, and premium loading factor m, given by π=mqs.^7^ Our model assumes countries pay insurance premiums from their own wealth. In practice, donor subsidies or aid might be available, but our model makes a simplifying assumption that any donor aid is eventually accessible to the country. Therefore, receiving a premium subsidy is essentially equivalent to reallocating the country’s existing wealth toward paying the insurance premiums.

### Counterfactual 2: Country has capacity for forecast-based early action

In the second counterfactual, the country already possesses the capacity for forecast-based early actions but may choose to purchase anticipatory insurance to partially or fully finance these actions if optimal. The country invests a total amount c in forecast-based actions, of which s is financed by anticipatory insurance. Here, the decision-maker selects total anticipatory action investment c, optimal insurance coverage s, and payout frequency 1/q to maximize utility $U^{\left(2\right)}$:

(3) $$U^{\left(2\right)}=(1-q-r)\cdot u(w-\pi )+(q+r-p)\cdot u(w-\pi +s)\;\;+r\cdot u\left(w-\pi -l\right)+\left(p-r\right)\cdot u\left(w-\pi -l-c+\beta c+s\right),$$

where π=mqs and s≤c.^8^

In the first counterfactual, anticipatory insurance provides value through both capacity building—enabling forecast-based actions—and insurance-funded financing. Without both components, the country undertakes no anticipatory action. In the second counterfactual, the value derives solely from the insurance mechanism, since the country already has capacity and can invest in anticipatory actions independently. Availability of anticipatory insurance allows countries to augment existing investments, spending a total of c, with insurance funding s of this total.

Evaluating optimal coverage s and resulting utility under these two counterfactuals across varying economic parameters (e.g., payout frequency, basis risk, premium loading) helps address two questions. First, under what economic conditions is purchasing anticipatory insurance optimal? Second, under what conditions is the insurance financing mechanism relatively more valuable than capacity-building support? Before presenting results, we discuss key model parameter assumptions.

### Model parameters for numerical analysis

The optimization problem in Eq. 2 for the first counterfactual involves two choice variables, s and 1/q, while the one in Eq. 3 involves three choice variables, s,c, and 1/q. The two optimization routines do not yield analytically tractable solutions. So, we solve the country’s optimization problem numerically under a set of assumptions.

We make the following model assumptions for the economic analysis of the anticipatory insurance. The country faces a 1-in-5 year drought risk, i.e., p=0.20. We assume that the response cost is 50% of the available financial resources, i.e., w=0.5l. We model the drought probability and severity to be consistent with the evidence in literature and assumptions in ARC’s product design. Existing literature suggests that moderate to severe droughts occur roughly every 4 to 5 years (Thurlow et al. 2009; Kaluba et al. 2017; SADRI 2021a). ARC also estimates a drought occurrence frequency at every 4 years for Zambia and 5 years for Malawi (ARC 2023). Existing literature on the impact of a drought suggests that a severe drought can reduce agricultural production and income by 50% (Devereux 2007; McCarthy et al. 2021; Clarke and Ruth Vargas 2013). We relax these assumptions and test the sensitivity of our main results to a wide range of drought probability in Sect. 3.6.

The decision-maker mirrors the risk preferences of the country’s residents, who have constant relative risk-averse preferences over wealth with a relative risk aversion of γ. This means that the decision-maker derives a utility of $u(x)=\frac{w^{1-\gamma }}{1-\gamma }$ from a wealth level of w in period T=2. For our main analysis, we assume γ=2.

We also make assumptions about the skill of forecast technology. Our measure of forecast skill is the correlation between the occurrence of drought and the triggering of anticipatory insurance payments (or anticipatory actions) in our model. We allow this correlation to vary from 0.2 to 0.8, i.e., ρ∈[0.2,0.8], capturing a reasonably wide range of forecast skill. Prior studies suggest that subseasonal precipitation forecasts for Sub-Saharan Africa generally have lower skill, especially at lead times exceeding 2 weeks (de Andrade et al. 2021). Seasonal forecasts for dry Dec–Feb season have shown to have high reliability in Southern Africa (Weisheimer and Palmer 2014), and they are able to correctly indicate a below-normal rainfall season more than 50% of the time in parts of Africa (Novella and Thiaw 2016; IRI 2025). However, forecasts generally outperform climatology, particularly demonstrating higher skill for predicting below- or above-normal precipitation compared to near-normal conditions (Pirret et al. 2020). We consider a broader range of correlation (up to 0.8) to ensure our results remain valid under potential future improvements in forecasting.^9^ Although we present results across a wide range of forecast skills, the current skill remains relatively low. Therefore, policy implications should be interpreted keeping in mind existing forecast capabilities.

We assume that the benefit-to-cost ratio of early actions varies in the range of 1 to 2, i.e., β∈[1,2]. The empirical evidence on the benefits of forecast-based actions is scarce. We rely on secondary survey results and empirical studies that estimate the benefits of early drought interventions (Clarke and Ruth Vargas 2013; Arndt et al. 2016; Walls et al. 2023). Appendix Sect. A.1 provides a detailed review of the existing literature. Finally, we consider two values of the premium loading—(a) the actuarially fair premium with m=1, and (b) m=1.35, i.e., the anticipatory insurance premium is 1.35 times the actuarially fair price (the expected loss).

### What is the optimal anticipatory insurance coverage?

#### When a country lacks the early action capacity

Figure 5 shows optimal insurance coverage for a country lacking early-action capacity, varying across basis risk (ρ), anticipatory action return period (1/q), premium loading (m), and benefit-to-cost ratio (β). The x-axis represents the anticipatory action return period (1/q), and the y-axis represents coverage s as a proportion of loss l. The figure contains 16 panels, varying basis risk (correlation between drought occurrence and insurance payout) along rows and benefit-to-cost ratios along columns. Solid and dashed black lines indicate optimal coverage at premium loadings of 0% and 35%, respectively.

Figure 5 shows that with high forecast skill (low basis risk, top row) and zero premium loading, optimal anticipatory coverage equals the maximum early-action level required to fully recover potential losses, i.e., l/β.^10^ However, higher premium loadings or basis risk typically reduce optimal coverage below this maximum. Optimal coverage also increases with the insurance payout return period, suggesting that lower-frequency (higher return period) payouts support higher optimal coverage. For instance, in the second row’s first panel (β=1.25,ρ=0.6), optimal coverage is approximately 0.6 for a 5-year return period but nearly 0.8 at over 20 years. These results indicate that anticipatory insurance is valuable for countries lacking self-financing capacity, providing both funds and early-action capacity. Even under unfavorable conditions—such as a 5-year return period, a low correlation (0.20), and modest early-action returns (1.25)—optimal coverage remains positive. Next, we examine optimal coverage for countries capable of independently undertaking anticipatory actions.

### When a country has the early action capacity

In this scenario, we assume a country already has the capacity to execute forecast-based actions independently, operating an existing early-action mechanism and optimally financing these actions before considering anticipatory insurance. Here, anticipatory insurance primarily provides additional financing to augment early actions when adverse events are forecasted.

Figure 4 shows optimal insurance coverage across different values of basis risk, return periods, premium loadings, and benefit-to-cost ratios of early actions. The solid and dashed black lines represent optimal ceding ratios at premium loadings of 0% and 35%, respectively. The solid red line indicates the total investment c in anticipatory actions, including both self-funded and insurance-funded components (s).^11^

**Fig. 3:**
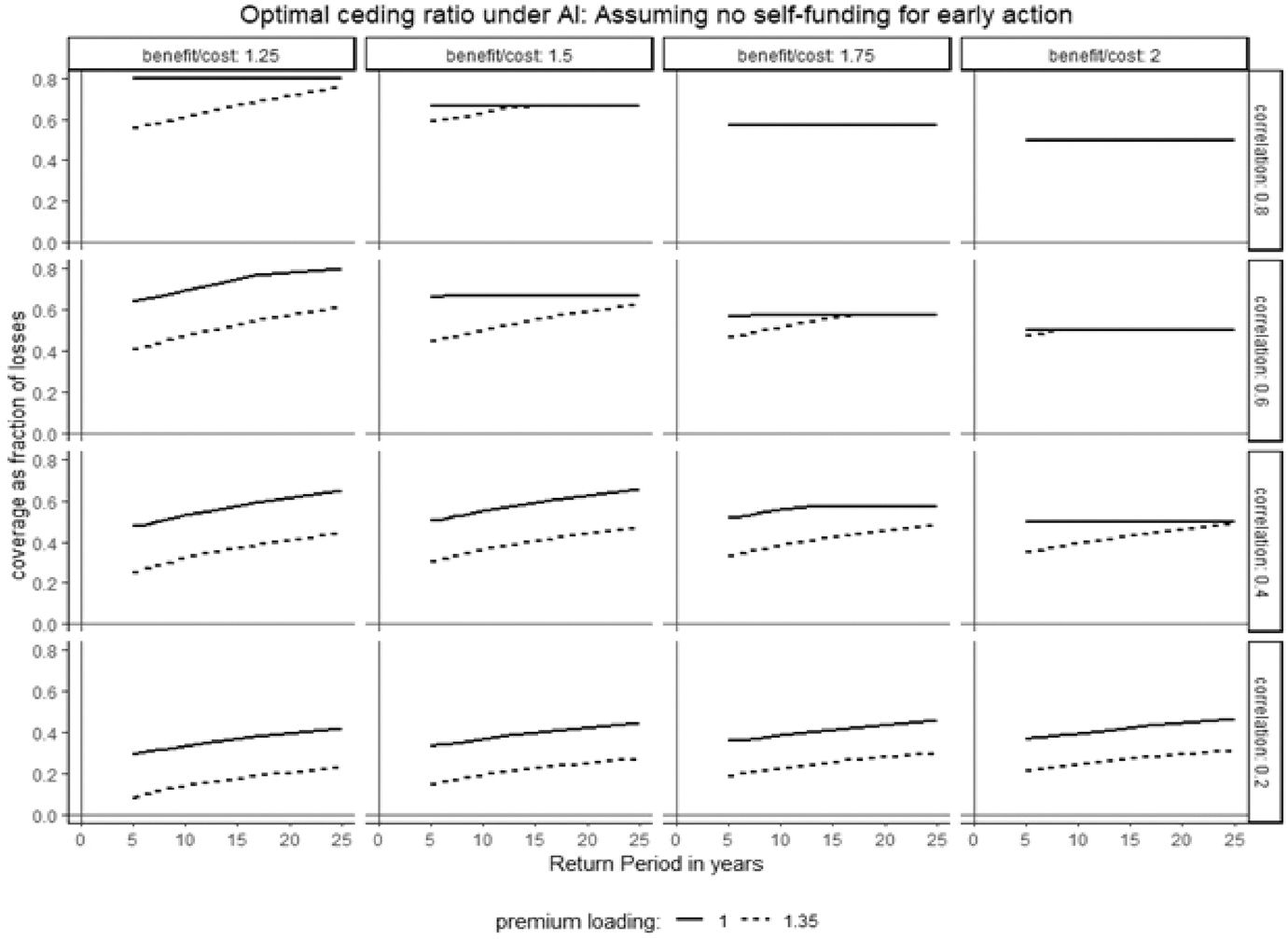
Optimal coverage assuming no self-finance early action. *Note* the figure plots the optimal ceding ratio (fraction of response cost covered by anticipatory insurance) under varying basis risk, return periods, premium loadings, and benefit-to-cost ratios of early actions, assuming no self-financing of forecast-based actions by the country. The *x*-axis shows the return period of the covered loss event; the *y*-axis shows the ceding ratio. The 16 panels vary basis risk (correlation between drought occurrence and anticipatory insurance payout) along rows, and benefit-to-cost ratios along columns. Solid and dashed lines represent optimal ceding ratios at premium loadings of 0% and 35%, respectively. Analysis assumes relative risk aversion of 2 and total response cost equal to 50% of the country’s wealth

**Fig. 4:**
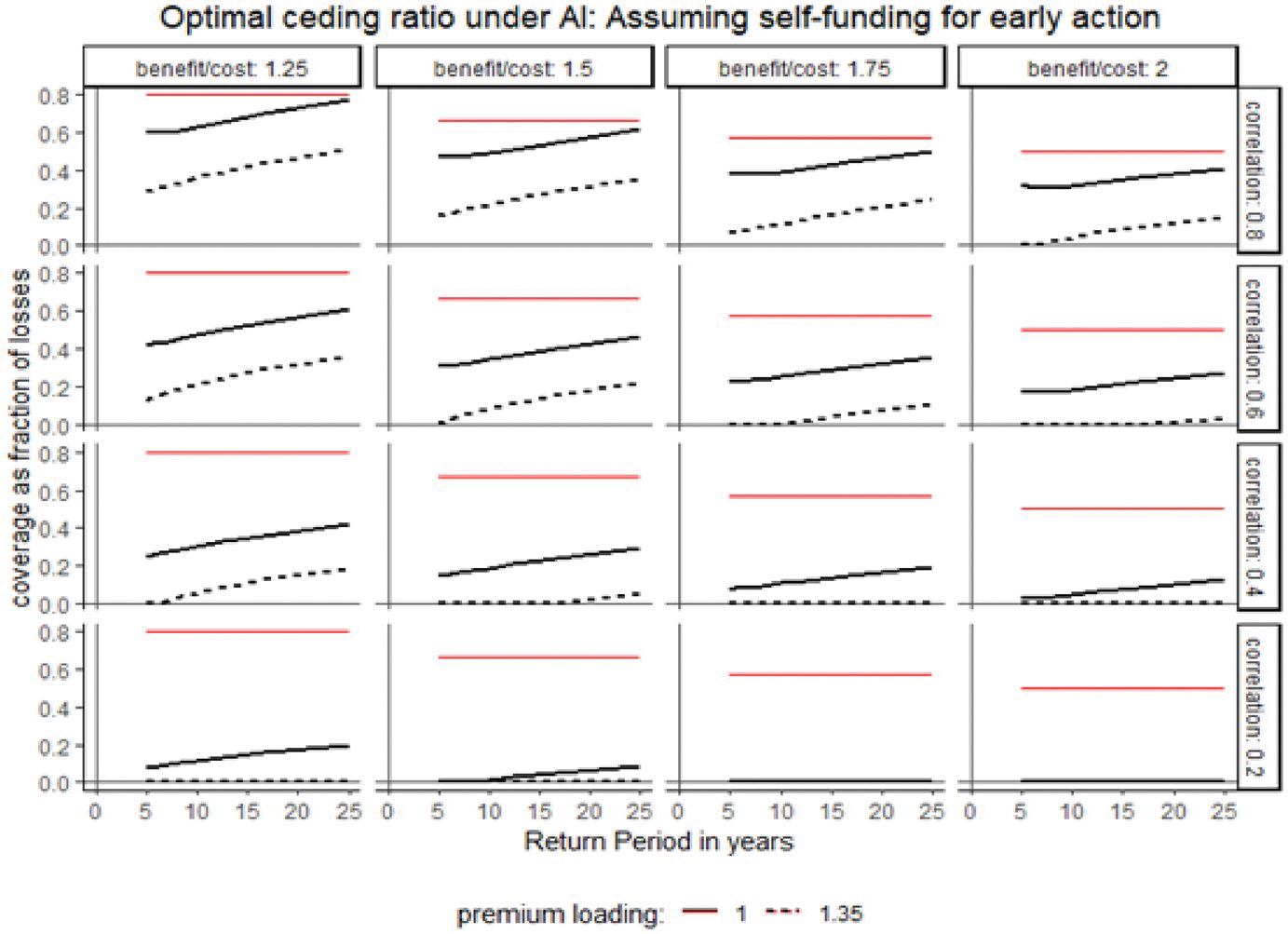
Optimal coverage assuming self-financing for early action. *Note* the figure plots optimal ceding ratios (fraction of response cost covered by anticipatory insurance) under varying basis risk, return periods, premium loadings, and benefit-to-cost ratios of early actions, assuming the country can independently execute forecast-based early actions. The *x*-axis represents the return period of the covered loss event; the *y*-axis represents the ceding ratio. The 16 panels vary basis risk (correlation between drought occurrence and insurance payout) along rows and benefit-to-cost ratios along columns. Solid and dashed black lines indicate optimal ceding ratios for premium loadings of 0% and 35%, respectively. The solid red line shows total anticipatory action investment (self-funded plus insurance-funded). The analysis assumes relative risk aversion of 2 and total response costs equal to 50% of the country’s wealth. (Color figure online)

The key insight is that when countries already have forecast-based early action mechanisms and can self-finance them, purchasing anticipatory insurance is not always optimal. Optimal insurance coverage exceeds zero only if basis risk, premium loadings, or early-action benefit-to-cost ratios fall below certain thresholds. Figure 4 shows that optimal coverage decreases as premium loadings or basis risk increase (moving top to bottom along a column) and as the benefit-to-cost ratio increases (moving left to right along a row). Although initially counterintuitive, the decrease in optimal coverage with higher benefit-to-cost ratios follows from reduced total optimal early-action investment.^12^ As early-action effectiveness (β) increases, required total investment declines, reducing demand for insurance coverage. Additionally, optimal coverage is sensitive to premium loadings. At a 35% premium loading (135% of actuarially fair pricing), optimal coverage is negligible when basis risk and early-action effectiveness are high-contrasting results from the first counterfactual (Fig. 3). This occurs because, with existing self-financing capacity, paying high premium loadings under elevated basis risk is less advantageous than directly investing in highly effective early actions.

### What is the welfare addition from anticipatory insurance?

**Fig. 5:**
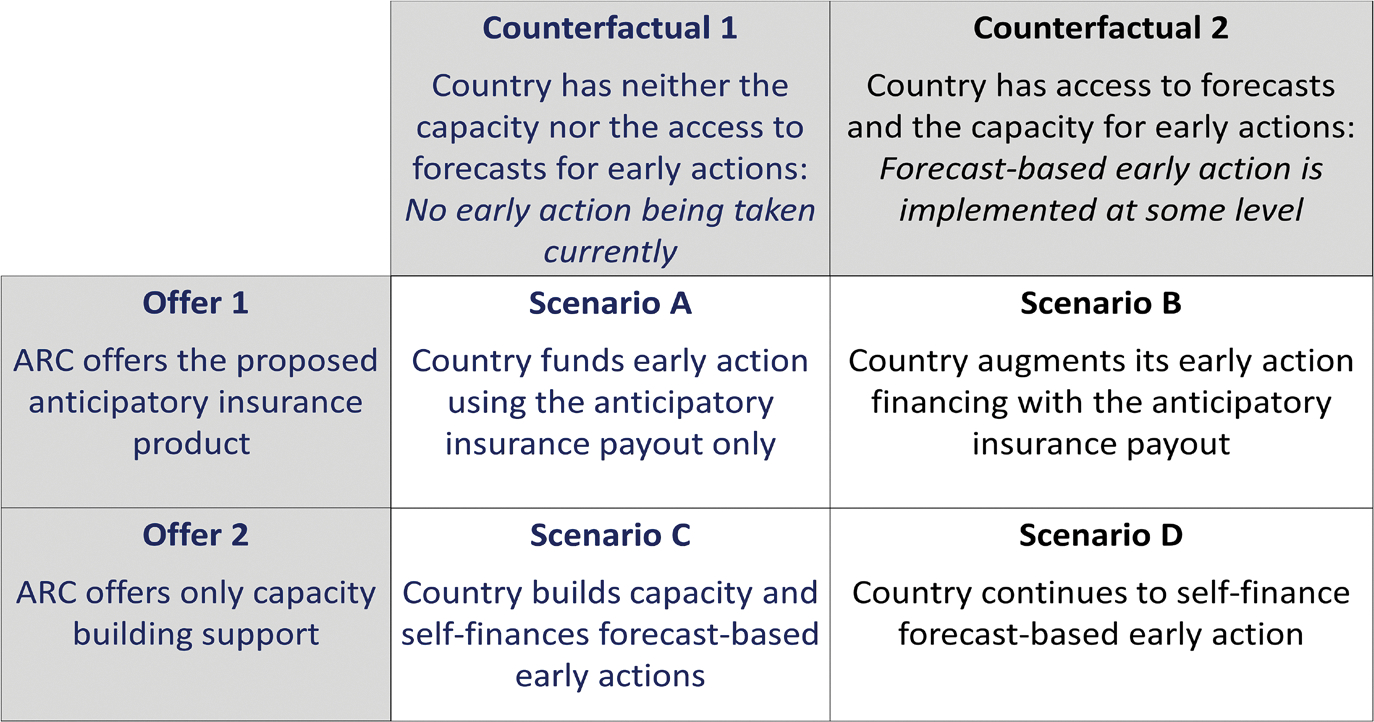
Alternative risk-financing strategies under different counterfactuals. *Note* the figure shows different scenarios based on two products offered by ARC and two counterfactuals (based on current state of anticipatory action capacity available to countries). Offer 1 is the proposed anticipatory insurance. Offer 2 includes only the capacity-building support with no insurance mechanism. Counterfactual 1 is when a country does not have capacity to take forecast-based anticipatory action in the absence of the anticipatory insurance. Counterfactual 2 is when a country has the capacity to take anticipatory action on its own

In this section, we examine how much better or worse off a country is using anticipatory insurance compared to relying solely on capacity-building support and self-financing anticipatory actions. Welfare is measured by certainty-equivalent wealth—the guaranteed amount of wealth making the country indifferent between that amount and facing uncertain losses managed by a particular risk-financing strategy. Using the two previous counterfactual scenarios (country with or without existing early-action capacity), we analyze two product types from ARC: the anticipatory insurance providing both capacity-building support and forecast-based payouts, and an alternative offering only capacity-building support (without insurance payouts). The latter allows countries to build early-action capacity and use forecasts while relying on self-financing (direct aid or budget allocations). Combining the two counterfactual country capacities with the two product offerings results in four scenarios, summarized in Fig. 5.

We focus on two comparisons. First, we compare welfare gains between Scenario A (no existing early-action capacity) and Scenario B (existing capacity), to assess the additional welfare provided by anticipatory insurance to countries without early-action capacity. This helps identify locations and economic conditions where anticipatory insurance is most beneficial. Second, within the first counterfactual scenario (no early-action capacity), we compare anticipatory insurance to a product offering only capacity-building support. This isolates the value added by the insurance mechanism, revealing when standalone capacity-building support may be sufficient versus when bundling with anticipatory insurance adds greater value.

#### Scenario A vs. B: Welfare gain from the anticipatory insurance based on a country’s early action capacity

In this section, we examine additional welfare provided by anticipatory insurance under two counterfactuals: (CF1) the country lacks early-action capacity and takes no early actions; and (CF2) the country has existing early-action capacity and currently finances actions optimally without insurance. Most countries likely fall between these extremes. In CF1, anticipatory insurance adds value through forecast information, capacity-building support, and insurance-based financing. In CF2, its value arises solely from insurance-based financing.

**Fig. 6:**
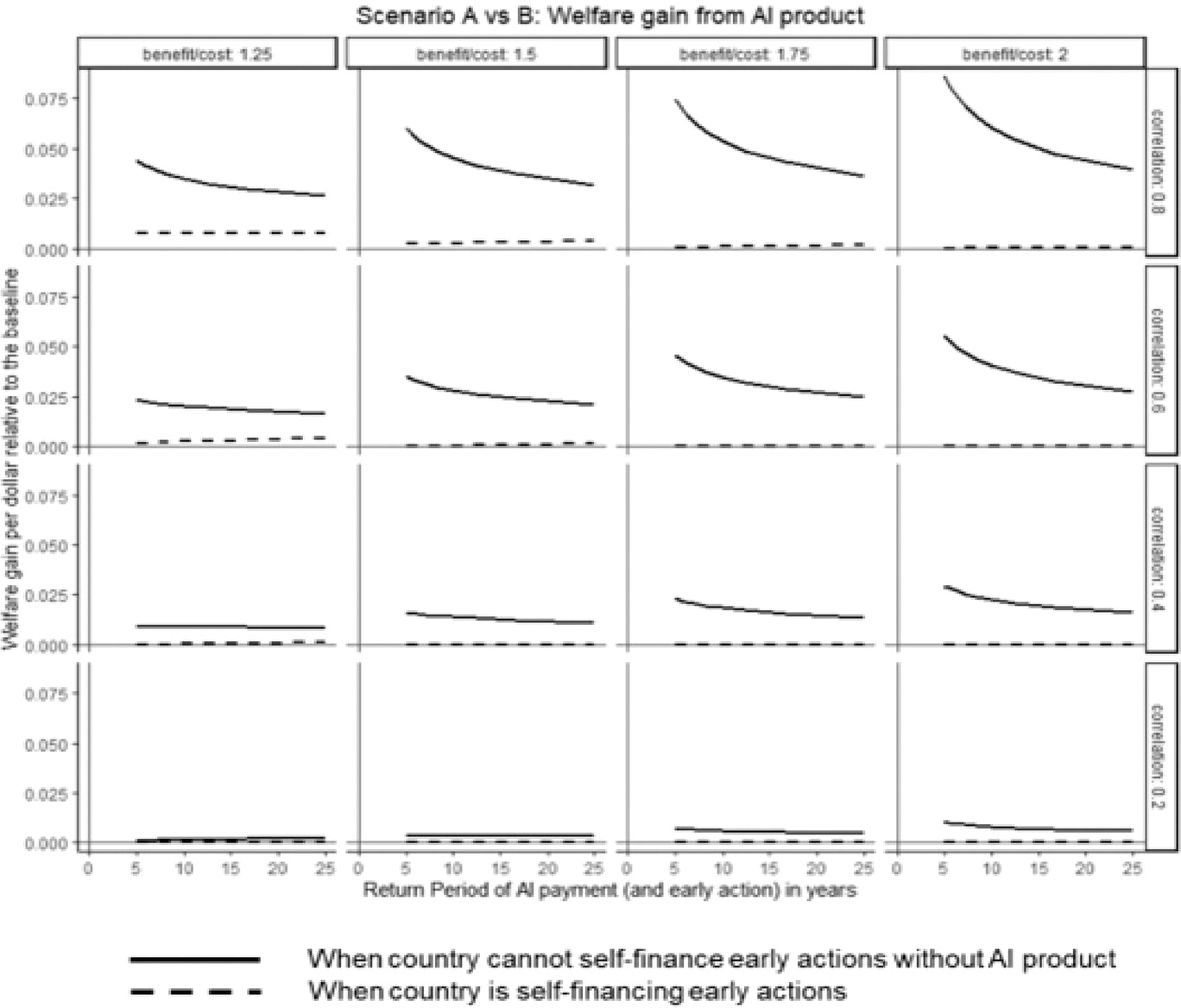
Welfare gain from anticipatory insurance: Scenario A vs. B. *Note* the figure shows welfare gains from anticipatory insurance (AI) under two counterfactuals across varying basis risk, return periods, premium loading, and benefit-to-cost ratios of early actions. Solid lines represent counterfactual 1 (country cannot self-finance early actions), dashed lines represent counterfactual 2 (country can self-finance). The *x*-axis indicates the return period of anticipatory insurance payouts; the *y*-axis shows welfare gains as a fraction of baseline welfare. The 16 panels vary basis risk (correlation between drought occurrence and insurance payout) along rows and benefit-to-cost ratios along columns. Analysis assumes 35% premium loading, relative risk aversion of 2, and total response cost equal to 50% of the country’s wealth

Figure 6 plots welfare gains from anticipatory insurance (as a fraction of baseline welfare) under two counterfactuals, varying basis risk, return period, premium loading, and early-action benefit-to-cost ratio. Solid lines represent Scenario A (no self-financing capacity) and dashed lines Scenario B (self-financing capacity). Baseline welfare is the certainty equivalent without anticipatory insurance under each scenario. The x-axis shows the insurance payout return period; the y-axis shows fractional welfare gains, assuming a premium loading of 35%.

There are three key insights. First, anticipatory insurance provides greater value when countries lack early-action capacity; the solid line (Scenario A) always lies above the dashed line (Scenario B), regardless of basis risk, return period, or benefit-to-cost ratio. Second, when countries can self-finance anticipatory actions, welfare gains from insurance are modest, indicating the main benefit of anticipatory insurance arises from building early-action capacity rather than insurance-based financing. Third, solid lines slope downward, while dashed lines slope slightly upward, implying that without self-financing capacity, frequent insurance payouts (shorter return periods) significantly increase welfare gains. Conversely, when self-financing is possible, less frequent payouts (longer return periods) slightly enhance welfare gains, since frequent payouts offer limited incremental benefit relative to insurance premiums.

#### Scenario A vs. C: Should we bundle anticipatory insurance with capacity building support?

Figure 6 shows that anticipatory insurance primarily benefits countries by providing early-action capacity-building support. What if the insurance component were unbundled from the anticipatory product, offering capacity-building support alone? Here, we compare welfare gains from two ARC products (see Fig. 5): (1) anticipatory insurance linking early-action plans directly to insurance payouts, and (2) a hypothetical standalone capacity-building support without insurance financing. Both products enable forecast-based early actions, but the first finances these actions exclusively through insurance payouts, while the second develops capacity allowing countries to self-finance early actions (using their own resources).^13^

**Fig. 7:**
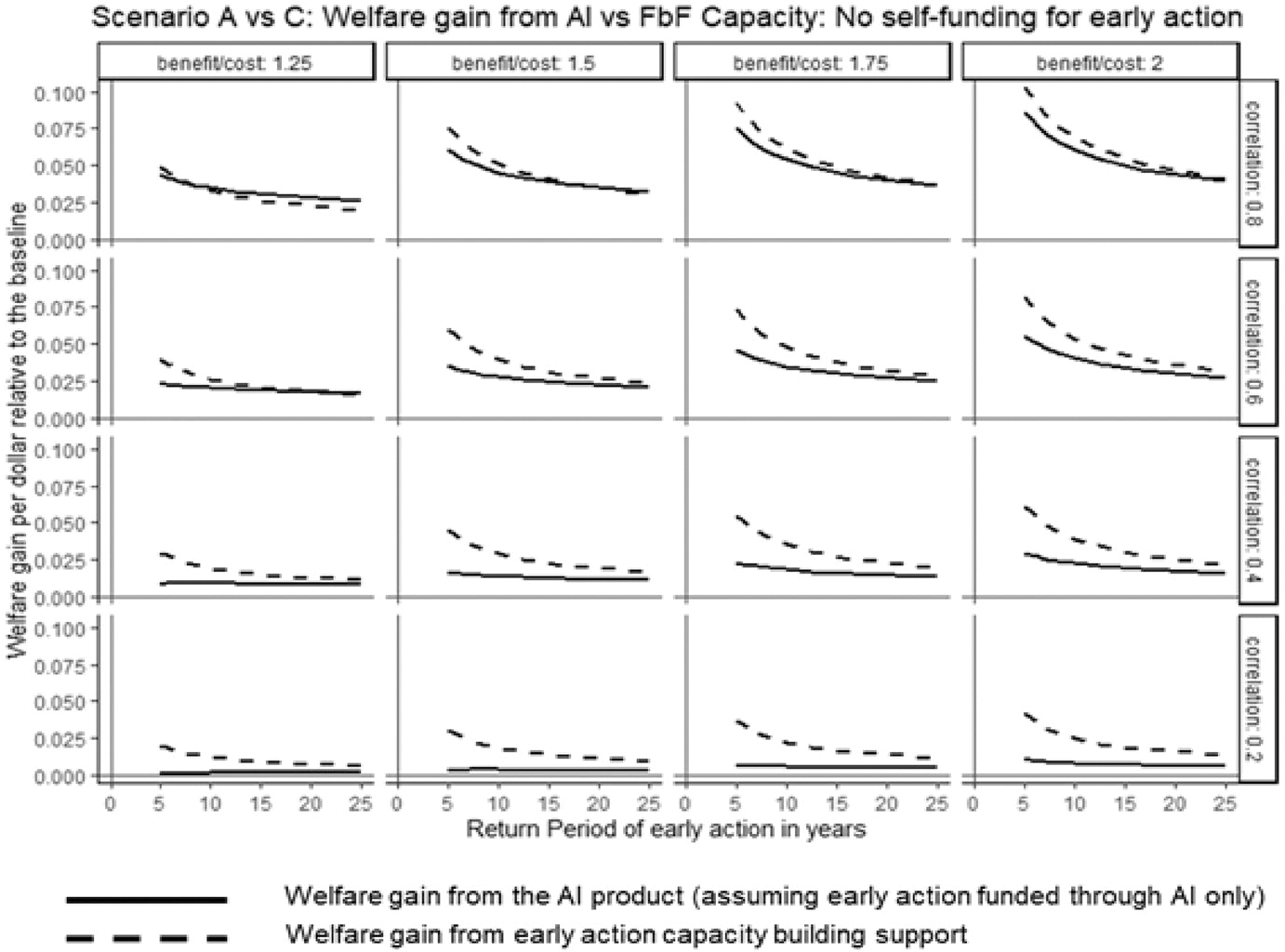
Welfare gains from capacity building vs. anticipatory insurance: Scenario A vs. C. *Note* the figure compares welfare gains from anticipatory insurance (solid line) and standalone capacity-building support (dashed line), assuming no existing anticipatory actions. Welfare gains (*y*-axis) are expressed as fractions of baseline welfare, across varying return periods (*x*-axis), basis risk levels (rows), and benefit-to-cost ratios (columns). Analysis assumes premium loading of 35%, relative risk aversion of 2, and total response cost equal to 50% of the country’s wealth

Figure 7 compares welfare gains from anticipatory insurance (solid line) and pure capacity-building support (dashed line) for countries lacking early-action capacity. Baseline assumes no forecast-based actions. The x-axis shows the return period of anticipatory insurance or early-action financing; the y-axis shows welfare gains as fractions of baseline welfare. The premium loading is 35%. The figure indicates that pure capacity-building support generally offers greater benefits, as its welfare gains (dashed line) exceed those of anticipatory insurance (solid line) in most conditions. Anticipatory insurance only outperforms pure capacity building when the benefit-to-cost ratio is low and forecast accuracy is high (low basis risk), where insurance financing provides additional value due to costly early actions. The advantage of pure capacity-building support grows as basis risk or early-action frequency increases. Under moderately low basis risk and infrequent actions (longer return periods), both products yield similar gains, whereas higher basis risk consistently favors pure capacity-building support.

### Sensitivity analysis

In this section, we examine the sensitivity of our main results to variations in the probability and severity of drought events. In the primary analysis, we estimated the optimal coverage for anticipatory insurance (Fig. 4) and its relative value compared to pure capacity-building services for anticipatory action (Fig. 7) for a drought with a probability of 0.20 and a severity translating to a response cost of 50% of disposable wealth. We re-estimated these results across a range of drought probability and severity scenarios based on prior research.

Previous literature has extensively examined drought probabilities and severities in Sub-Saharan Africa, particularly focusing on Zambia and Malawi. Precisely defining drought events is challenging, which complicates accurate estimations of their likelihood and severity. Both academic and policy literature estimate drought return periods between 4 and 5 years in the region, translating to probabilities of 20% to 25% (SADRI 2021a, b). We adopt a drought probability of 20% in our primary analysis, aligning with average probabilities from earlier research. However, existing studies indicate that drought frequency may rise due to decreasing precipitation in the region (Mtilatila et al. 2020). Additionally, drought return periods vary by severity and geography. Prior research estimates probabilities ranging from 33 to 11% for moderate droughts and 20% to below 2% for severe droughts across different regions (Kaluba et al. 2017).^14^

To determine a relevant range of drought severity, we reviewed literature quantifying drought impacts on national and regional economies. Existing studies suggest severe droughts can reduce agricultural production and income by 30–45% (Devereux 2007; Clarke and Ruth Vargas 2013). Economic impacts range from minor local effects to significant national disruptions, affecting various sectors. For example, Tembo et al. (2020) estimate that droughts can cause up to a 60% decline in Zambia’s manufacturing sector, representing approximately 7% of the GDP. Given that a substantial proportion of the population in Zambia and Malawi relies on small-scale agriculture, the primary impact channel for these countries is likely agriculture (Thurlow et al. 2009). This reliance on rainfed agriculture heightens economic vulnerability to droughts. In Zambia, over half of maize cultivation is rainfed (Ngoma et al. 2021). Crop yields in Sub-Saharan Africa significantly fluctuate with rainfall variability (Thurlow et al. 2009; Coughlan de Perez et al. 2019). Drought conditions, for instance, can result in yield declines between 30 and 80% (McCarthy et al. 2021).

To account for a wide range of drought probability and severity, we vary the drought probability from 0.02 (once in 50 years) to 0.33 (once in 3 years) and drought severity from a 10% reduction in wealth to a 50% reduction in wealth.^15^ We test the sensitivity of our two key results to all combination of these probability–severity pairs. First, we estimate the sensitivity of optimal coverage under anticipatory insurance from Fig. 4. Second, we estimate the sensitivity of the incremental welfare gain from anticipatory insurance over a pure capacity-building support, i.e., the difference in solid line over the dashed line from Fig. 7. In order to present our sensitivity results in a way that is easy to comprehend, we replicate our analysis to the anticipatory insurance that has a premium loading of 35% and an attachment point such that it pays every 25 years. We choose this higher return period to show a sensitivity analysis for the upper limit of the product’s benefits since the anticipatory insurance coverage and relative welfare gains increase with return period as shown in Figs. 4 and 7, respectively.

### Sensitivity analysis: optimal coverage under anticipatory insurance

**Fig. 8:**
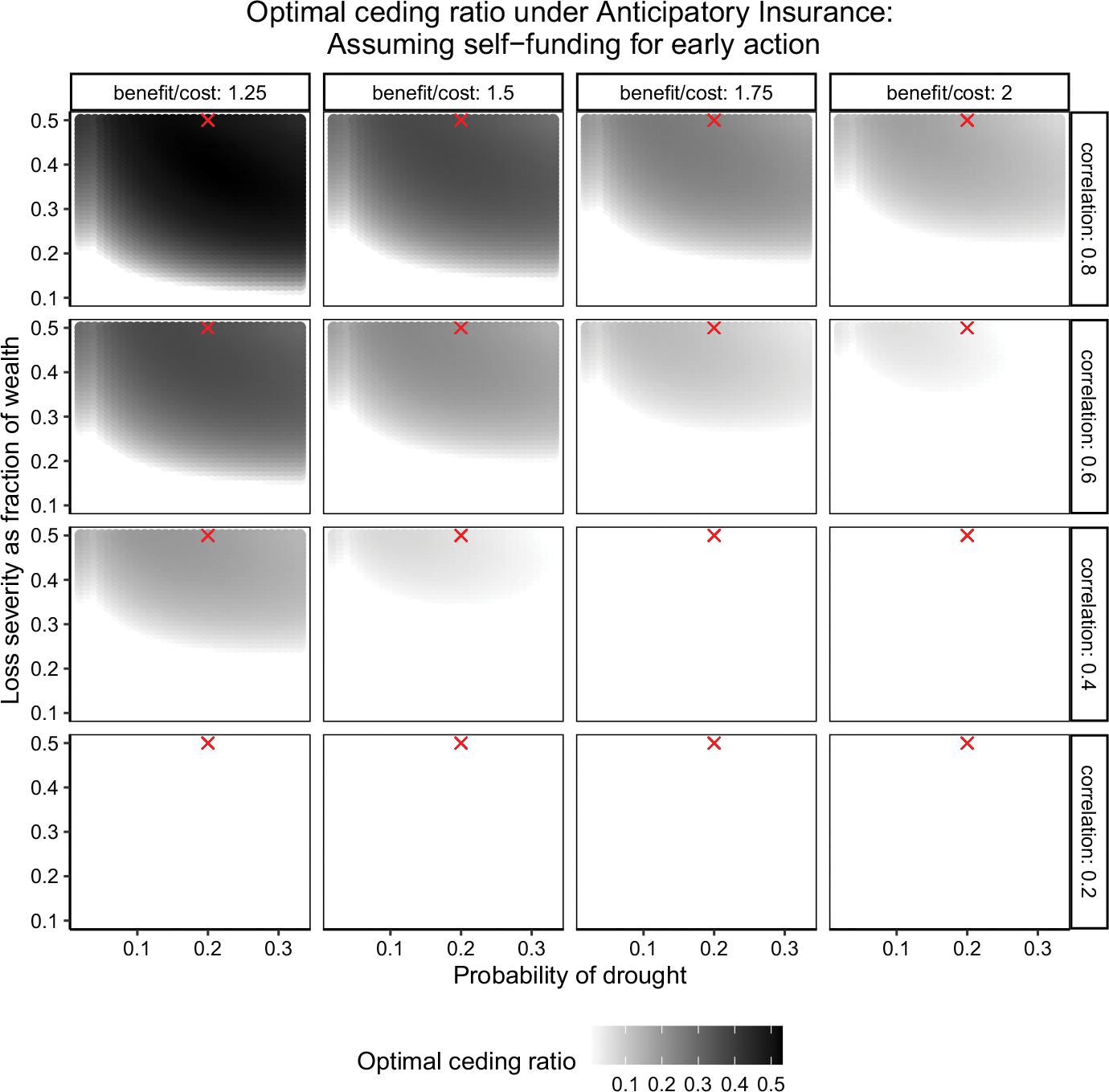
Sensitivity of optimal coverage assuming self-financing for early action. *Note* the figure plots the optimal ceding ratio of the anticipatory insurance for different values drought probability (*x*-axis), severity (*y*-axis), basis risk (row panels) and benefit-to-cost ratio (column panels) of early actions. The analysis assumes a scenario where the country has capacity to take forecast-based early actions in the absence of the anticipatory insurance. The shaded area denotes that optimal coverage is positive, and a darker shade represents a higher coverage. The red crosses are probability and severity used in main analysis results shown in Fig. 4. The return period for insurance payout is 25 years and the premium loadings is 35%. The analysis assumes a relative risk aversion of 2. (Color figure online)

Figure 8 plots the result of the sensitivity analysis for optimal coverage under anticipatory insurance when a country also can also self-finance early action. Specifically, this figure replicates the coverage with return period of 25 years and premium loading of 35% from Fig. 4 for different values of drought probability and severity. In Fig. 8, each panel shows the optimal anticipatory insurance coverage by drought probability (x-axis) and severity (y-axis). There are 16 panels that show how this sensitivity varies with basis risk (by row) and benefit-to-cost ratio of early actions (by column). The shaded area denotes that optimal coverage is positive, and a darker shade represents a higher coverage. The red marker on each graph denotes the drought probability of 0.20 and severity of 0.5 (the response cost-to-wealth ratio), which are used in our main results in Fig. 4. For example, the top-left panel plots the optimal insurance coverage as a fraction of response cost for a scenario with correlation between forecast and drought occurrence is 0.8 (lowest basis risk) and benefit-to-cost ratio of early action is 1.25.

Figure 8 shows that optimal coverage is positive and higher when basis risk is lower (correlation is higher). Furthermore, the optimal coverage increases as benefit-to-cost ratio of early actions decreases. These results are consistent with those shown in Fig. 4. Sensitivity analysis also shows that, in general, coverage is higher for more severe droughts. For example, when the correlation is 0.4 and the benefit-to-cost ratio is 1.25, the optimal coverage is positive only for droughts that result in a response cost of at least 25% of the country’s wealth. Conditional on basis risk, benefits-to-cost ratio of actions, and drought severity, there does not appear to be a significant effect of drought probability on optimal coverage.

### Sensitivity analysis: relative welfare gain from anticipatory insurance

**Fig. 9:**
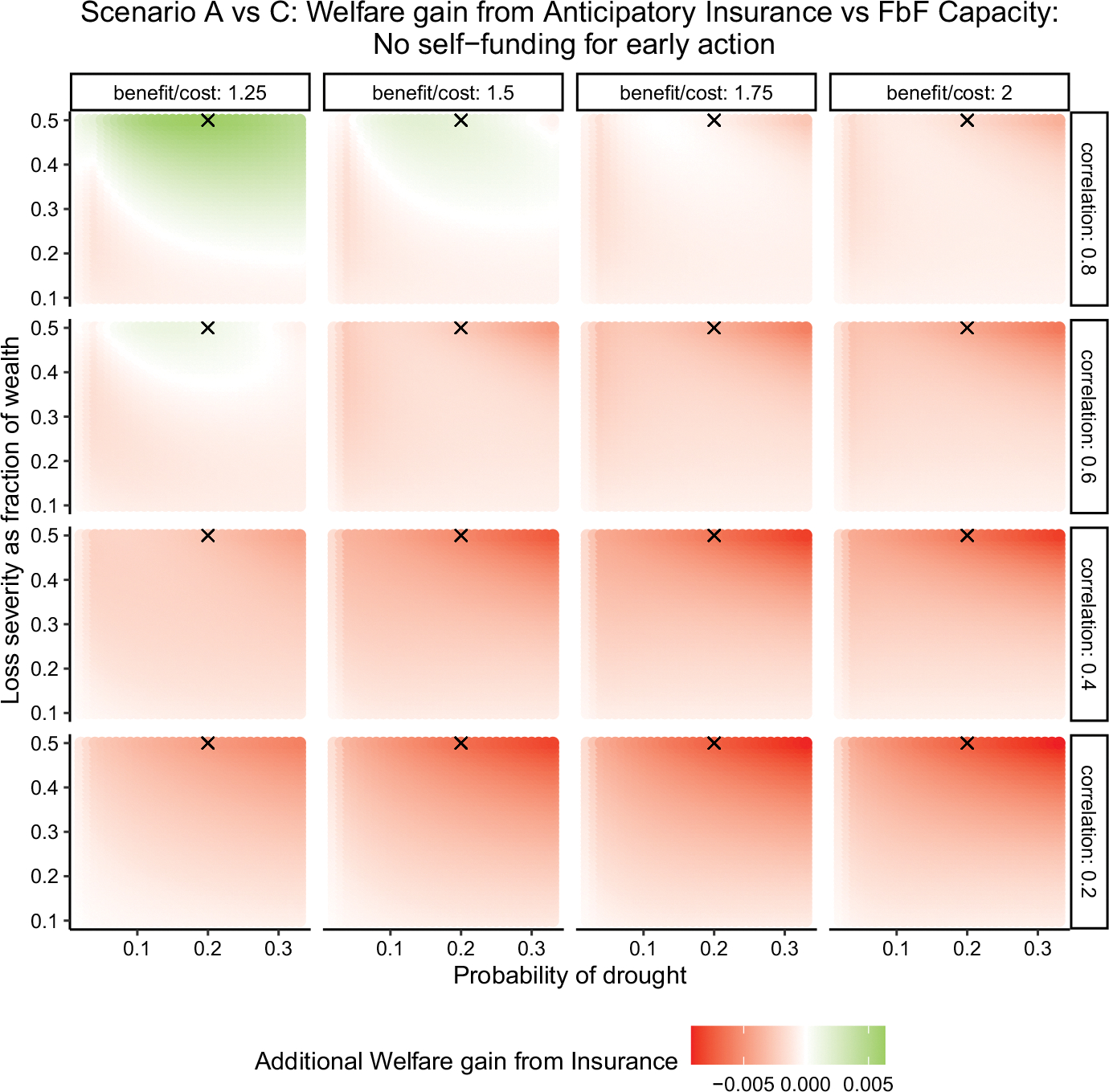
Sensitivity of welfare gain from anticipatory insurance. *Note* the figure plots the welfare gain from anticipatory insurance relative to a pure early action capacity-building service for different values drought probability (*x*-axis), severity (*y*-axis), basis risk (row panels) and benefit-to-cost ratio (column panels) of early actions. The green shaded area denotes a positive welfare gain, the white shade denotes zero welfare gain, and the red shade denotes a negative welfare gain. The black markers on each graph denote the drought probability of 0.20 and severity of 0.5 (the response cost-to-wealth ratio), which are used in our main results in Fig. 7. The return period for insurance payout is 25 years and the premium loadings is 35%. The analysis assumes a relative risk aversion of 2. (Color figure online)

Figure 9 plots the result of the sensitivity analysis for welfare gain from anticipatory insurance relative to a pure early action capacity-building service. Specifically, this figure replicates the finding from Fig. 7 for different values of drought probability and severity. The results are replicated for anticipatory insurance with a payment return period of 25 years and premium loading of 35%. In Fig. 9, each panel shows the relative welfare gain from anticipatory insurance by drought probability (x-axis) and severity (y-axis). Rest of the figure format is same as in Fig. 8. The green shaded area denotes a positive welfare gain, the white shade denotes zero welfare gain, and the red shade denotes a negative welfare gain. The black markers on each graph denote the drought probability of 0.20 and severity of 0.5 (the response cost-to-wealth ratio), which are used in our main results in Fig. 7.

Figure 9 shows that relative welfare gain from anticipatory insurance is positive only when both the basis risk and the benefit-to-cost ratio of early actions are lower, i.e., the top-left panel of the figure. These results are consistent with those shown in Fig. 7. Sensitivity analysis also shows that, within these requirements on basis risk and benefit–cost ratio of actions, relative welfare gain is higher for more severe and more frequent droughts. These results suggest that our results and recommendations on when anticipatory insurance is most valuable do not vary significantly with drought probability and severity.

### Key inferences from economic analysis

The demand for anticipatory insurance is always positive when a country lacks the capacity for forecast-based early actions. However, when early-action capacity exists, anticipatory insurance is optimal only if basis risk, premium loadings, or benefit-to-cost ratios are sufficiently low.

Countries with existing early-action capacity face two trade-offs. The first is between investing in early actions now or using resources for post-disaster recovery, which becomes more challenging when early-action benefits are low relative to costs. Spending more on early actions increases marginal costs. The second trade-off involves insurance: paying premiums risks having fewer resources if the disaster occurs without triggering an insurance payout (basis risk). This risk is particularly severe when basis risk is high. However, when basis risk is low and early-action benefits are modest relative to costs, anticipatory insurance allows countries to finance early actions without fully exhausting current resources. Thus, insurance financing is most beneficial under low basis risk and relatively costly early actions, enabling countries to fund actions they could not otherwise afford.

In most other cases, the primary value of anticipatory insurance stems from its capacity-building support and provision of forecast information, rather than its insurance mechanism. Hence, the choice between anticipatory insurance and standalone capacity-building support depends mainly on basis risk and early-action effectiveness.

**Fig. 10:**
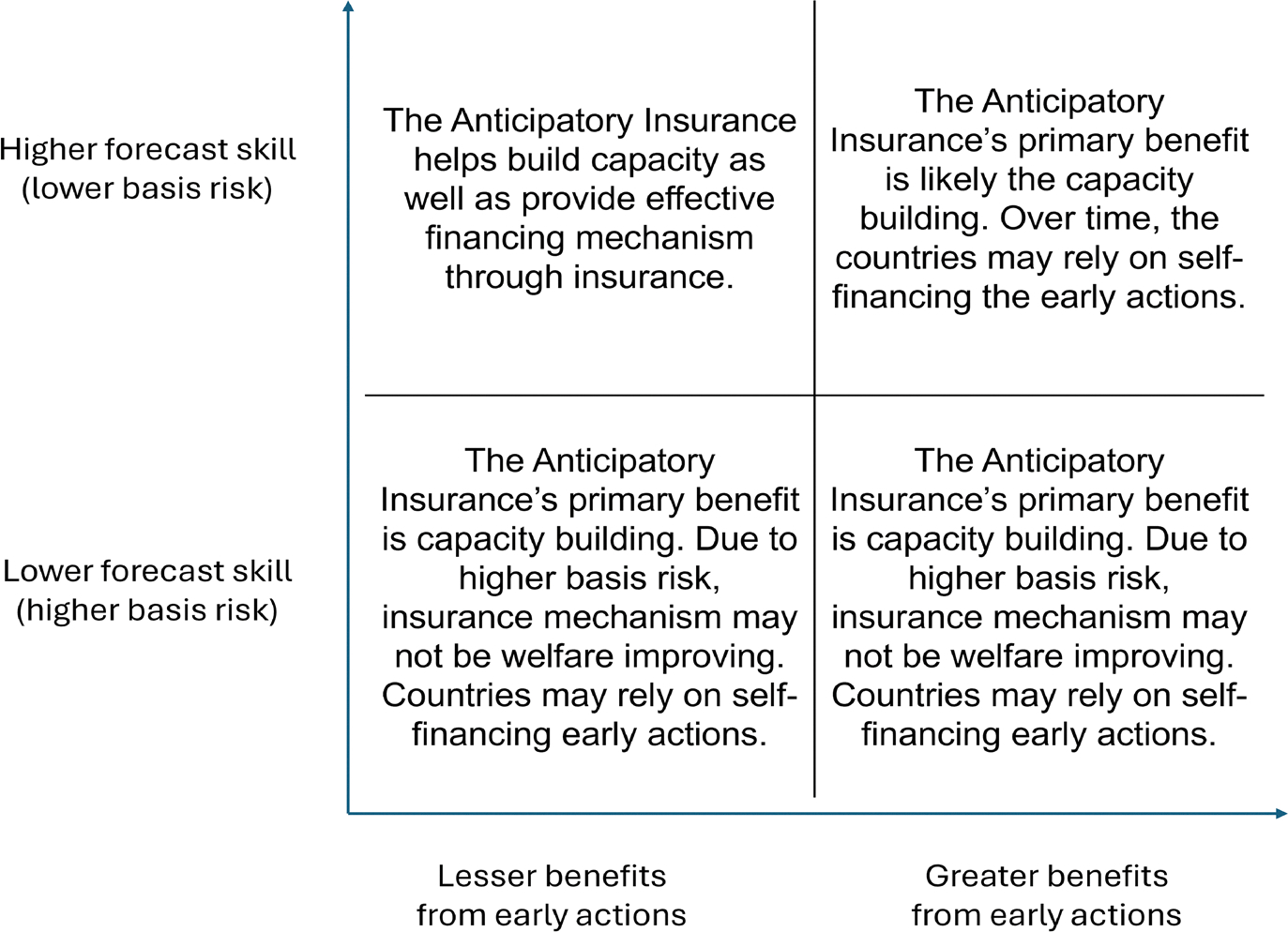
When is anticipatory insurance valuable? *Note* the figure shows the primary benefit of an anticipatory insurance mechanism based on the forecast skill and available early actions for a country that otherwise does not have the capacity to take anticipatory actions

Overall, these results indicate that capacity building and forecast access are essential for effective forecast-based early actions, while insurance financing provides secondary benefits. Countries with limited or no existing early-action capacity may struggle to redirect resources toward building such capacity. Here, anticipatory insurance can encourage policymakers and stakeholders to develop the necessary infrastructure and processes. Once sufficient early-action capacity exists, countries can choose financing sources based on forecast accuracy and action effectiveness. Figure 10 summarizes these findings for countries without early-action capacity. ARC should therefore first assess a country’s current early-action capacity and the quality of forecasts and early actions available, to determine the optimal balance of capacity-building support and anticipatory insurance in the short and long term.

### Qualitative study

We complement our economic analysis with a qualitative study involving document reviews and stakeholder interviews to deepen understanding of the anticipatory insurance product and validate our modeling assumptions.

### Methodology

We conducted a detailed review of approximately 50 ARC-provided documents, including technical reports, policy documents, contingency plans, insurance agreements, risk assessments, and forecasting outputs. This review established an initial understanding of anticipatory insurance, its processes, and fund-access mechanisms. From this, we developed a preliminary Theory of Change (ToC) outlining assumptions critical for successfully implementing the product and achieving its benefits. This ToC was first reviewed by stakeholders selected jointly with ARC and the research team. It was then refined further through feedback gathered during ARC workshops held in Malawi and Zambia. Figure 2 shows a simplified ToC for anticipatory insurance implementation in Malawi.

We also conducted in-depth semi-structured interviews with 10 stakeholders selected through snowball sampling, including local government officials, representatives from UN’s OCHA and ARC, and a local farmer. Individualized interview guides were developed to address gaps identified during the document review and to capture diverse perspectives on program objectives, key assumptions, challenges, and potential improvements. Key interview insights are summarized in the following section.^16^

### Key insights

Interviews highlight that anticipatory insurance in Malawi depends critically on accurate forecasts, effective stakeholder coordination, and overcoming logistical constraints. This aligns with assumptions in our economic model and theory of change (Fig. 2). Interviewees identify coordination as an additional challenge beyond forecast accuracy and logistical issues, reinforcing prior evidence (Ruesch et al. 2021) that stakeholder alignment is critical. Thus, insurance financing alone is insufficient for effective anticipatory action. Specifically, the interviews highlight three key insights.

First, climate change is changing farming conditions, prompting stakeholders to value anticipatory actions. A less intuitive advantage is increased preparedness enthusiasm among adopters. Yet success hinges on accurate forecasts and rapid implementation. Slow distribution risks farmers being unable to use resources timely within the season, causing unnecessary payouts (“triggering in vain”) and potentially making anticipatory insurance less advantageous than traditional insurance, which pays after drought confirmation. Second, effective anticipatory action requires coordination and timely consultation among at-risk populations, local governments, humanitarian actors, and other stakeholders. Interviewees emphasized information dissemination, quick procurement, and political support as essential for effective fund and resource transfers. This aligns with our economic model emphasis on capacity building for long-term effectiveness. Third, despite challenges, anticipatory insurance can help farmers adapt to drought, reducing negative coping behaviors such as selling productive assets. Early payouts may allow replanting or diversifying income strategies (Coughlan de Perez et al. 2024). Given that many countries have existing anticipatory programs, this product can complement and strengthen current institutional structures.

In summary, qualitative findings underline capacity building as central to anticipatory insurance effectiveness. While insurance financing is valuable, it represents only a part of the solution. The other important component is establishing robust institutional arrangements—accurate forecasts, efficient procurement, stakeholder coordination, and clear communication channels—that enable timely anticipatory actions from policymakers to end-beneficiaries.

## Conclusion

Forecast-contingent insurance is an innovative mechanism to fund early actions for disaster risk management. We evaluate a drought anticipatory insurance developed by a leading provider, identifying conditions under which it adds value. This product offers capacity building and forecast-based financing for early actions. Our analysis provides two key insights. First, anticipatory insurance primarily benefits countries without existing early-action capacity, with most welfare gains driven by capacity building rather than insurance payouts. Optimal demand is generally positive only if capacity is lacking, or when existing capacity yields relatively low-benefit actions and forecasts are highly accurate—making it more beneficial to purchase index insurance instead of self-financing less effective early actions. Second, the core value of anticipatory insurance product comes from its capacity-building component. Providing standalone capacity-building support may often be more beneficial, as insurance premium costs can exceed the incremental value of insurance payouts.

Overall, these results suggest that forecast-based risk mitigation may benefit more from capacity building and forecast access than insurance financing. However, many existing programs lack the comprehensive setup needed for timely anticipatory actions, making anticipatory insurance valuable for short-term capacity development. This is particularly true when allocating funds for early actions faces challenges due to uncertainty, anticipatory insurance’s combined financing and capacity building can demonstrate benefits clearly, fostering long-term political support for forecast-based interventions.

## Supplementary Material

Online Appendix

**Supplementary Information** The online version of this article (https://doi.org/10.1057/s41288-025-00355-2) contains supplementary material, which is available to authorized users.

## Figures and Tables

**Table 1:** Joint probabilities of insurance payout and drought occurrence

	No anticipatory insurance payout	Anticipatory insurance payout

No Drought	1−q−r	q+r−p
Drought	r	p−r

The table shows the joint probabilities of drought and anticipatory insurance payout. p is the unconditional probability of drought happening in a year. q is the probability that forecast probability of drought exceeds the trigger and the insurance payout is triggered, i.e., $q=\text{Prob}(\phi \geq \phi_{\text{att}}$). r, which denotes basis risk, is the probability that drought occurs and insurance does not pay

## Data Availability

No empirical used.

## Footnotes

1 As of August 2024, forecast-based early action programs exist in 43 countries, with 119 active plans addressing 12 hazard types. These target more than 17 million people with around USD 260 million invested. In addition, 83 frameworks are being developed in 62 countries (German Red Cross 2024).

2 Although countries might have some capability for early actions, their systems may not be sufficient for time-sensitive forecast-based actions. Therefore, capacity-building support may be valuable even when an early action framework exists. For countries without access to forecasts, anticipatory insurance can also facilitate obtaining forecast information.

3 For forecast-based early action programs to be effective, countries need institutional and operational capacity to act quickly once a forecast is available. This includes institutional coordination, clear standard operating procedures, awareness of vulnerable areas and communities, trained workers, established procurement agreements, and allocated funds for early actions.

4 The updated probability may be thought of as the probability of drought conditional on available information about future weather in the country. e.g., the drought index output from the proprietary model, or the El Nino forecast.

5 The model assumes that the primary purpose of the anticipatory actions is to prevent losses due to drought. For example, early actions during a drought can prevent households from selling productive assets to finance food and essential requirements. Many actions such as providing drought-resistant seeds and tubers are geared to prevent higher losses during drought. However, during good states of economy, we assume there is neither a penalty nor any benefit from taking early actions. Similarly, in an extreme case when the losses are fully recovered through early actions, any additional action does not provide any incremental return.

6 A model that addresses this limitation will ideally model drought probability and response cost jointly. Specifically, it will model the joint distribution of drought severity l and probability p(l), by making the probability a function of the severity. In such model, the insurance payouts will be modeled as a function of severity l and not a choice variable.

7 The actuarially fair premium (m=1) equals qs.

8 We simplify by assuming self-funded and insurance-funded actions share the same forecast trigger frequency (1/q). Although theoretically distinct triggers are possible, practical considerations favor consistency and coordination.

9 While weather forecasts have gradually improved over time, recent applications of deep learning methods have accelerated these advancements (Lam 2023).

10 For example, if anticipatory actions yield $1.25 per $1 spent (top-left panel), spending l/1.25 results in a benefit equal to l, fully covering losses. The y-axis coverage fraction is thus 1∕1.25 = 0.8.

11 The optimal total anticipatory action investment, c, fully recovers all losses. Total investment c is independent of forecast skill (Anand 2022) and return period, due to a fixed assumed return on early actions. Thus, the red line remains flat, determined solely by the benefit-to-cost ratio.

12 The optimal total investment in early actions is proportional to 1/β.

13 In practice, even under anticipatory insurance, countries might eventually self-finance early actions once capacity is built. However, short-term extension of insurance-linked operational plans for self-financing can be costly.

14 For instance, Kaluba et al. (2017) suggest that a moderate drought—defined as rainfall below 80% of mean annual precipitation—has return periods varying from 3 years (33% annual probability) in the South to 9 years (11%) in the North, where as a severe droughts, defined as rainfall below 60% of mean annual precipitation, have return periods ranging from 5 years (20%) to approximately 55 years (below 2%).

15 Our range of probability also corresponds to empirically validated extremely dry conditions with cumulative probability of nearly 2% to mildly dry conditions with cumulative probability of nearly 30% (NASA 2025).

16 Detailed interview quotes and thematic summaries are provided in “Appendix” Table 2.

## References

[R1] Aguirre Julio, De La Torre Daniel, Bazo Ugarte Juan, Quequezana Paulo, and Collado Mauricio. 2019. Evaluation of early action mechanisms in Peru regarding preparedness for El Niño. International Journal of Disaster Risk Science 10 (4): 493–510.

[R2] Anand Vaibhav. 2022. Forecast-based financing for risk mitigation. SSRN Working Paper. https://papers.ssrn.com/abstract=5211250.

[R3] Anand Vaibhav. 2025. Does getting forecasts earlier matter? Evidence from winter advisories and vehicle crashes. American Economic Journal: Economic Policy. 10.2139/ssrn.4206910.

[R4] ARC. 2023. Technical note on anticipatory action insurance model. Johannesburg: African Risk Capacity.

[R5] Arndt Channing, Pauw Karl, and Thurlow James. 2016. The economy-wide impacts and risks of Malawi’s Farm Input Subsidy Program. American Journal of Agricultural Economics 98 (3): 962–980.

[R6] Barnett Barry J., and Mahul Olivier. 2007. Weather index insurance for agriculture and rural areas in lower-income countries. American Journal of Agricultural Economics 89 (5): 1241–1247.

[R7] Bauer Peter, Thorpe Alan, and Brunet Gilbert. 2015. The quiet revolution of numerical weather prediction. Nature 525 (7567): 47–55. 26333465 10.1038/nature14956

[R8] Braman Lisette Martine, Krispijn van Aalst Maarten, Mason Simon J., Suarez Pablo, Ait-Chellouche Youcef, and Tall Arame. 2013. Climate forecasts in disaster management: Red Cross flood operations in West Africa, 2008. Disasters 37 (1): 144–164. 23066755 10.1111/j.1467-7717.2012.01297.x

[R9] Burlig Fiona, Jina Amir, Kelley Erin M., Lane Gregory V., and Sahai Harshil. 2024. Long-range forecasts as climate adaptation: Experimental evidence from developing-country agriculture. NBER Working Paper 32173.

[R10] Chantarat Sommarat, Mude Andrew G., Barrett Christopher B., and Carter Michael R.. 2013. Designing index-based livestock insurance for managing asset risk in northern Kenya. Journal of Risk and Insurance 80 (1): 205–237.

[R11] Clarke Daniel J. 2016. A theory of rational demand for index insurance. American Economic Journal: Microeconomics 8 (1): 283–306.

[R12] Clarke Daniel J., and Dercon Stefan. 2016. Dull disasters? How planning ahead will make a difference. Washington, DC: World Bank.

[R13] Clarke Daniel, and Hill Ruth Vargas. 2013. Cost–benefit analysis of the African Risk Capacity Facility. IFPRI Discussion Paper 1292. Washington, DC: International Food Policy Research Institute (IFPRI).

[R14] Cole Shawn, Xavier Giné Jeremy Tobacman, Topalova Petia, Townsend Robert, and Vickery James. 2013. Barriers to household risk management: Evidence from India. American Economic Journal: Applied Economics 5 (1): 104–135. 24765234 10.1257/app.5.1.104 PMC3995033

[R15] Collier Benjamin L. 2020. Strengthening local credit markets through lender-level index insurance. Journal of Risk and Insurance 87 (2): 319–349.

[R16] Collier Benjamin, Skees Jerry, and Barnett Barry. 2009. Weather index insurance and climate change: Opportunities and challenges in lower income countries. The Geneva Papers on Risk and Insurance: Issues and Practice 34 (3): 401–424.

[R17] de Andrade FM, Young MP, MacLeod D, Hirons LC, Woolnough SJ, and Black E. 2021. Subseasonal precipitation prediction for Africa: Forecast evaluation and sources of predictability. Weather and Forecasting 36 (1): 265–284.

[R18] Coughlan de Perez Erin, van den Hurk B, Van Aalst MK, Jongman Brenden, Klose T, and Suarez Pablo. 2015. Forecast-based financing: an approach for catalyzing humanitarian action based on extreme weather and climate forecasts. Natural Hazards and Earth System Sciences 15 (4): 895–904.

[R19] Coughlan de Perez Erin, van Aalst Maarten, Choularton Richard, van den Hurk Bart, Mason Simon, Hannah Nissan, and Saroja Schwager. 2019. From rain to famine: assessing the utility of rainfall observations and seasonal forecasts to anticipate food insecurity in East Africa. Food security 11 (1): 57–68.

[R20] Coughlan de Perez Erin, Anderson Weston, Han Eunjin, Gibbon Innocent Tirivanhu Masukwedza, and Ntlele Mphonyane 2024. Detectable use of ENSO information on crop production in Southern Africa. Climate services 36: 100514. 39717387 10.1016/j.cliser.2024.100514 PMC11666189

[R21] Devereux Stephen. 2007. The impact of droughts and floods on food security and policy options to alleviate negative effects. Agricultural Economics 37 (s1): 47–58.

[R22] Ehrlich Isaac, and Becker Gary S.. 1972. Market insurance, self-insurance, and self-protection. Journal of Political Economy 80 (4): 623–648.

[R23] German Red Cross. 2024. Anticipatory action at a glance. German Red Cross. https://www.anticipation-hub.org/experience/global-map. Accessed 1 Sep 2024.

[R24] Giné Xavier, Townsend Robert, and Vickery James. 2008. Patterns of rainfall insurance participation in rural India. World Bank Economic Review 22 (3): 539–566.

[R25] Gros Clemens, Pronti Andrea, Sheikh Khairul, Hassan Ahmadul, and Shahjahan Mohammad. 2023. Effects of anticipatory humanitarian cash assistance to households forecasted to experience extreme flooding: Evidence from Bangladesh. Hydrology Research 54 (11): 1315–1328.

[R26] IPCC. 2021. Summary for policymakers. In Climate change 2021: The physical science basis. Contribution of Working Group I to the Sixth Assessment Report of the IPCC, 3–32. Cambridge: Cambridge University Press.

[R27] IRI. 2025. Seasonal forecast verification. Palisades: Columbia University: International Research Institute for Climate and Society.

[R28] Kaluba P, Verbist KMJ, Cornelis WM, and Van Ranst E. 2017. Spatial mapping of drought in Zambia using regional frequency analysis. Hydrological Sciences Journal 62 (11): 1825–1839.

[R29] Khalil Abedalrazq F., Hyun-Han Kwon, Upmanu Lall, Mario J Miranda, and Jerry Skees. 2007. El Niño-Southern Oscillation-based index insurance for floods: Statistical risk analyses and application to Peru. Water Resources Research. 10.1029/2006WR005281.

[R30] Lala Jonathan, Bazo Juan, Anand Vaibhav, and Block Paul. 2021. Optimizing forecast-based actions for extreme rainfall events. Climate Risk Management 34 : 100374.

[R31] Lam R, 2023. Learning skillful medium-range global weather forecasting. Science 382:1416–1421. 37962497 10.1126/science.adi2336

[R32] Linnerooth-Bayer Joanne, Mechler Reinhard, and Pflug Georg. 2005. Refocusing disaster aid. Science 309 (5737): 1044–1046. 16099976 10.1126/science.1116783

[R33] Linsenmeier Manuel, and Shrader Jeffrey G.. 2023. Global inequalities in weather forecasts. Charlottesville: Center for Open Science.

[R34] Lopez Ana, Coughlan de Perez Erin, Bazo Juan, Suarez Pablo, van den Hurk Bart, and van Aalst Marteen. 2020. Bridging forecast verification and humanitarian decisions: A valuation approach for setting up action-oriented early warnings. Weather and Climate Extremes 27: 100167.

[R35] McCarthy Nancy, Kilic Talip, Brubaker Josh, Murray Siobhan, and de la Fuente Alejandro. 2021. Droughts and floods in Malawi: Impacts on crop production and the performance of sustainable land management practices under weather extremes. Environment and Development Economics 26 (5–6): 432–449.

[R36] Molina Renato, and Rudik Ivan. 2022. The social value of predicting hurricanes. SSRN Working Paper 4266614. 10.2139/ssrn.4266614.

[R37] Mortensen Eric, and Block Paul. 2018. ENSO index-based insurance for agricultural protection in southern Peru. Geosciences 8 (2): 64.

[R38] Mtilatila Lucy, Bronstert Axel, Gerd Bürger, and Klaus Vormoor. 2020. Meteorological and hydrological drought assessment in Lake Malawi and Shire River Basins (1970–2013). Hydrological Sciences Journal 65 (16): 2750–2764.

[R39] NASA. n.d. Standardized precipitation index (SPI). Washington, DC: NASA. Accessed 3 April 2025.

[R40] Ngoma Hambulo, Lupiya Patrick, Kabisa Mulako, and Hartley Faaiqa. 2021. Impacts of climate change on agriculture and household welfare in Zambia: An economy-wide analysis. Climatic Change 167 (3): 55.

[R41] Novella Nicholas S., and Thiaw Wassila M.. 2016. A seasonal rainfall performance probability tool for famine early warning systems. Journal of Applied Meteorology and Climatology 55 (11): 2575–2586.

[R42] OCHA (ReliefWeb). 2023. Upscaling and risk-layering for climate resilience in Malawi. New York: OCHA.

[R43] Pirret JSR, Daron JD, Bett PE, Fournier N, and Foamouhoue AK. 2020. Assessing the skill and reliability of seasonal climate forecasts in Sahelian West Africa. Weather and Forecasting 35:1035–1050.

[R44] Rosenzweig Mark R., and Udry Christopher R.. 2019. Assessing the benefits of long-run weather forecasting for the rural poor: Farmer investments and worker migration in a dynamic equilibrium model. w25894. Cambridge: National Bureau of Economic Research.

[R45] Ruesch Lea, Tarakci Murat, Besiou Maria, and Neils VanQuaquebeke. 2021. Orchestrating coordination among humanitarian organizations. Production and Operations Management 31 (5): 1977–1996.

[R46] SADRI. 2021a. Drought resilience profile, Zambia. Southern Africa Drought Resilience Initiative (SADRI).

[R47] SADRI. 2021b. Drought resilience profile, Zambia. Southern Africa Drought Resilience Initiative (SADRI).

[R48] Shrader Jeffrey. 2023. Improving climate damage estimates by accounting for adaptation. SSRN Scholarly Paper ID 3212073. Rochester: Social Science Research Network.

[R49] Teh Tse-Ling., and Christopher Woolnough. 2019. A better trigger: Indices for insurance. Journal of Risk and Insurance 86 (4): 861–885.

[R50] Tembo Bernard, Sihubwa Sydney, Masilokwa Ignatius, and Mulima Nyambe-Mubanga. 2020. Economic implications of climate change in Zambia. Working Paper 137. Pretoria: Southern Africa-Towards Inclusive Economic Development (SA-TIED).

[R51] Thurlow James, Zhu Tingju, and Diao Xinshen. 2009. The impact of climate variability and change on economic growth and poverty in Zambia. IFPRI Discussion Paper 00890. Washington, DC: International Food Policy Research Institute.

[R52] Walls Helen, Johnston Deborah, Matita Mirriam, Chirwa Ephraim, Mazalale Jacob, Quaife Matthew, Kamwanja Tayamika, and Smith Richard. 2023. How effectively might agricultural input subsidies improve nutrition? A case study of Malawi’s Farm Input Subsidy Programme (FISP). Food Security: The Science, Sociology and Economics of Food Production and Access to Food 15 (1): 21–39.

[R53] Weisheimer A, and Palmer TN. 2014. On the reliability of seasonal climate forecasts. Journal of the Royal Society of Interface 11 (96): 20131162. 10.1098/rsif.2013.1162 PMC4032526 24789559

[R54] World Bank. 2021. Benin Country Economic Memorandum. World Bank Country Economic Memorandum. Washington, DC: World Bank.

