# Supplementary material for: When does forecast-based insurance benefit? An economic analysis of drought risk anticipatory insurance
Source: Geneva Pap Risk Insur Issues Pract. Author manuscript; Available in PMC 2025 Oct 11. (PMC12512034; doi:10.1057/s41288-025-00355-2)
Supplement: Online Appendix [file NIHMS2112356-supplement-Online_Appendix.pdf]

Online Appendix

When does Forecast-based Insurance benefit?
An Economic Analysis of Drought Risk Anticipatory Insurance

A Appendix

A.1 Assumptions on cost-benefit ratio of early actions

Since ARC anticipatory insurance has not yet made payouts, we rely on secondary evidence to estimate plausible benefit-cost ratios assuming all Theory of Change conditions hold. A meta-analysis by Hemming et al. (2018) indicates that farm inputs consistently improve yields and incomes, with an average yield increase of 0.11 standard deviations. Malawi-specific studies align with these findings, showing improved maize adoption significantly raises household income, maize consumption, and asset value, especially among poorer farmers (Bezu et al. 2014; Tesfaye and Tirivayi 2020). Evidence on indirect benefits, such as dietary diversity or long-term child nutrition, is mixed. Malawi's farm subsidy program alone does not consistently improve dietary diversity (Walls et al. 2023; Matita et al. 2023), though seed diversity (e.g., legumes, sweet potatoes, cassava) may enhance dietary outcomes (Matita et al. 2022). Farm inputs alone typically have short-term impacts on child health, with long-term improvements dependent on combined maternal and community nutrition programs (Mwale et al. 2022).

Benefit-cost ratios from Malawi range from 0.76 to 2.3, typically around 1 to 2 (Arndt et al. 2015). Economy-wide estimates, accounting for indirect effects, can be higher (up to 1.99) due to diversification enabled by improved maize yields, though they are sensitive to marginal fertilizer returns. Importantly, drought-resistant seeds could maintain high yields in drought conditions, preserving these benefits even during rare droughts. For this analysis, we assume a benefit-cost ratio between 1 and 2.

A.2 Stakeholder Interview Observations

Table 2: Stakeholder Interview Observations

Sub-Theme	Summary	Selected Quote(s)	Implications
Theme: farmer context			
Climate change impacts	Climate change is affecting farming seasons, planting times, and changing the frequency and intensity of droughts.	"Due to climate change, the planting season has shifted to the end of October or November in some regions. Farmers sometimes have to wait until December for the onset of the rainy season"	How can attachment points for ARC consider the changing return periods of drought events? Can WRSI models properly represent both historical and current drought patterns?
Government support	Government subsidies, such as the Malawi Farm Input Subsidy Program, enable people to access essential inputs like seeds and fertilizer.	"Farmers often rely on the government for agricultural subsidies, particularly for essential inputs like seeds and fertilizers. When the government cannot provide these subsidies, it becomes challenging for farmers."	How will ARC inputs complement or replace existing farm subsidies from the government?
Farming has limited mechanization	Traditional farming practices are prevalent in Malawi, with a heavy reliance on manual labor, limited mechanization, and low pesticide use.	"Many farmers rely on manual labor often due to the lack of access to education and modern farming equipment, which is limited by financial constraints."	How are farm laborers that do not have decision making power on their farms included or excluded from the intervention? How is this reflected in targeting?

Continued on next page

Table 2 – *Continued from previous page*

Sub-Theme	Summary	Selected Quote(s)	Implications
Farmers have limited access to finance	Limited access to finance decreases agricultural productivity and economic development.	"The cost of farm inputs and limited access to finance are key factors affecting crop yields. Many farmers sell maize to afford seeds and fertilizer."	How does socioeconomic status impact utilization of the proposed intervention?
Theme: critical assumptions for success			
ARC will not be effective without consultation with at-risk populations	Need information dissemination about the insurance product to the public, coordination and collaboration among stakeholders, consultation with agriculture clusters, and needs assessments	"Dissemination of data is the challenge, most do not have access to the communication systems." "There also seemed to be a lack of scrutiny regarding the implementation details, as it appeared to be a policy-driven approach"	How will ARC engage with the public about this new product?
ARC will not be effective without government coordination	Collaboration with organizations like OCHA and the government is essential but faces challenges like lack of consultation and information sharing. Ensuring political buy-in and understanding of the program at all levels is important for successful implementation.	"There is a concern that the preparatory work might not be completed ahead of time due to limited government capacity. Therefore, the success of the process must rely on the individuals involved being driven by a commitment to doing good rather than on internal incentives or financial motivations." "The period from January to April is short and critically important for this process to succeed. It necessitates careful planning, coordination, and alignment of all stakeholders to ensure the efficient transfer of funds and resources to address the anticipated challenges."	How can ARC coordinate with the government and ensure confidence in the new product?

Continued on next page

Table 2 – *Continued from previous page*

Sub-Theme	Summary	Selected Quote(s)	Implications
Logistical constraints could limit the success of ARC	Success factors will include timely transfer of funds, effective targeting delivery and implementation logistics, availability of inputs, and efficient transfer of resources	"The transfer of funds needs to be done as quickly as possible, which is subject to the final implementation plan." "ARC needs to transfer the resources as quickly as possible, and obtain the right inputs on time so that farmers can make use of the remainder of the season."	How are benefits impacted if procurement of essential inputs fails? Can the Anticipatory Insurance product guarantee that farmers will receive inputs in time to replant?
Accurate forecasting will be required for success	Africa RiskView is instrumental in modeling the impact of droughts, and their forecasts will need to be accurate for the anticipatory insurance product to succeed.	"It's essential that by mid-January, we should be in a position where we are able to evaluate the status of the situation on the ground. A crucial assumption is that the forecasting model accurately matches the situation on the ground."	Do all stakeholders understand the magnitude of the basis risk?
ARC should evaluate success	Evaluation exercises are crucial to assessing the effectiveness of interventions and ensuring support reaches the right beneficiaries.	"Give room for independent evaluation for stakeholders to ensure that they are doing the right thing and that the support is going to the right people on the ground. Climatic shocks are here to stay and we can only improve on the implementation."	Can ARC do a rigorous enough evaluation to properly estimate the benefits after a payout?
Theme: anticipatory action could have positive impacts			

Continued on next page

Table 2 – *Continued from previous page*

Sub-Theme	Summary	Selected Quote(s)	Implications
ARC could help avoid sowing failure	Rather than waiting for the crops to fail, the Anticipatory Insurance payout would enable replanting and harvesting in what would have otherwise been a failed season.	"The historical drought is December to mid-January. In case of sowing failure around this time, the payout can be used from March to May. Payouts allow farmers to salvage part of the season rather than waiting for the end of the season. There are some crops and varieties that can be planted to salvage the season with residual moisture." "There would be an early payout so that farmers can make the best use of season."	Will the seeds and inputs produce sufficient yield on the timeline expected for the payout?
ARC could help prevent negative coping mechanisms	Maintaining farmer incomes can help people avoid negative coping mechanisms, such as the sale of productive assets, that have long-term negative consequences.	"Providing for immediate needs so that the household does not use negative coping mechanisms (consumption of foods that are not good foods, wild foods, households may revert to selling bicycles, radios and other assets in their households."	Is the expected benefit substantial enough to affect the use of negative coping mechanisms?
ARC could improve food security	With improved harvests during drought years, farmers and their families will have greater incomes and greater food consumption	"Responding early could elevate farmer wellbeing and reduce significantly the amount of finance needed to respond at the end of the season."	Will the provision of two types of crops (maize and tubers) improve dietary diversity?
